# Efficient fitting of single-crystal diffuse scattering in interaction space: a mean-field approach

**DOI:** 10.1107/S2052252521009982

**Published:** 2021-11-03

**Authors:** Ella M. Schmidt, Johnathan M. Bulled, Andrew L. Goodwin

**Affiliations:** a Inorganic Chemistry Laboratory, University of Oxford, South Parks Road, Oxford OX1 3QR, United Kingdom

**Keywords:** materials modelling, nanostructure, mean-field approach, diffuse scattering

## Abstract

The diffraction patterns of crystalline materials with strongly correlated disorder are characterized by the presence of structured diffuse scattering. Conventional analysis approaches generally seek to interpret this scattering either atomistically or in terms of pairwise (Warren–Cowley) correlation parameters. Here it is demonstrated how a mean-field methodology allows efficient fitting of diffuse scattering directly in terms of a microscopic interaction model. In this way the approach gives as its output the underlying physics responsible for correlated disorder. Moreover, the use of a very small number of parameters during fitting renders the approach surprisingly robust to data incompleteness, a particular advantage when seeking to interpret single-crystal diffuse scattering measured in complex sample environments. As the basis of this proof-of-concept study, a toy model is used based on strongly correlated disorder in diammine mercury(II) halides.

## Abstract 2

Mean-field theory is applied to model single-crystal diffuse scattering in molecular crystals with orientational disorder.

## 1. Introduction

Complex structures can emerge from simple interactions (Ziman, 1979 ▸; Parsonage & Staveley, 1978 ▸; Welberry, 1985 ▸). Geometric frustration in the Ising triangular antiferromagnet (Wannier, 1950 ▸), the hydrogen-bonding-driven configurational degeneracy of cubic ice (Bernal & Fowler, 1933 ▸) and the long-period stacking phases of the anisotropic next-nearest-neighbour interaction (ANNNI) model (Bak, 1982 ▸) are all well studied examples. It is a natural corollary that complexity is not particularly uncommon, and indeed there is a growing realization that complexity of various types is not only present but important for the behaviour of many key classes of functional materials, from disordered rocksalt cathodes to high-temperature superconductors (Clément *et al.*, 2020 ▸; Ji *et al.*, 2019 ▸; Mydosh & Oppeneer, 2011 ▸; Welberry & Goossens, 2016 ▸; Simonov & Goodwin, 2020 ▸). Determining the structures of such systems is one of the key challenges of modern structural science (Billinge & Levin, 2007 ▸; Keen & Goodwin, 2015 ▸; Juhás *et al.*, 2015 ▸).

**Figure 1:**
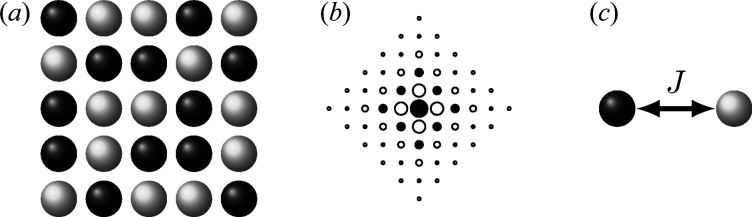
Descriptions of complex structures, illustrated for the case of a disordered binary alloy. (*a*) A representative atomistic configuration. (*b*) The auto-correlation function, with positive maxima represented by filled circles and negative maxima by empty circles. The size of the circle corresponds to the strength of the auto-correlation. (*c*) The interaction space driving the generation of atomistic models as in panel (*a*) and the corresponding auto-correlation function in panel (*b*).

Implicit in the term ‘complex’ is the inference that a very large number of parameters is needed to describe a structure meaningfully. In the case of disordered crystals, for example, one approach is to use atomistic configurations, each representing a structural fragment spanning sufficiently many unit cells to capture any key correlations; the corresponding number of descriptors is large indeed, because it scales with the supercell volume and is amplified further by the loss of crystal symmetry (McGreevy & Pusztai, 1988 ▸; Eremenko *et al.*, 2019 ▸; Goodwin, 2019 ▸) [Fig. 1 ▸(*a*)]. An alternative is to describe disordered structures in terms of interatomic correlations such as the Warren–Cowley parameters, since these are uniquely determined for disordered crystals even if they can be realized by many microscopically distinguishable configurations (Cowley, 1950 ▸; Weber & Simonov, 2012 ▸). Yet even these parameters are many because correlations usually extend across a large number of unit cells, there are many individual contributions to the correlation function for a given pair, and one needs in principle to consider not only two-body but also higher-order terms [Fig. 1 ▸(*b*)]. Hence there is an apparent paradox in that some complex structures can be succinctly generated, but not succinctly described.

An obvious resolution is to describe complex structures in terms of the (simple) interactions from which they arise – we will come to call this an ‘interaction space’ description. Doing so is the motivation, of course, of direct Monte Carlo (MC) studies of disordered and other complex materials: one tests candidate interaction models by comparing their predictions against observables, until the best model – the ‘solution’ – is identified (Weber, 2005 ▸; Welberry, 1985 ▸, 2004 ▸; Welberry *et al.*, 1998 ▸) [Fig. 1 ▸(*c*)]. In a crystallographic context, the key experimental observable one might use to best discriminate between different models is the structured diffuse scattering pattern measured in a suitable single-crystal diffraction experiment (Neder & Proffen, 2008 ▸; Welberry & Goossens, 2014 ▸). This diffuse scattering can be calculated directly from MC configurations, allowing a quantitative measure of the goodness of fit. A combination of (i) varying the interaction potentials and/or their descriptive parameter(s), (ii) re-running the corresponding MC simulations and (iii) assessing the change in quality of the fit to the data then forms the basis of an interaction-space refinement strategy, such as employed in the inverse Monte Carlo (IMC) (Weber, 2005 ▸; Almarza & Lomba, 2003 ▸; Jain *et al.*, 2006 ▸; D’Alessandro, 2011 ▸; Welberry, 2001 ▸) and empirical potential structure refinement (EPSR) (Soper, 1996 ▸, 2012 ▸) approaches.

Here we explore the viability of a particularly efficient alternative method for refining interaction parameters against diffuse scattering data that bypasses altogether the need to generate atomistic configurations *en route* [Fig. 1 ▸(*c*)]. Not only is the approach computationally attractive, but it removes the uncertainty introduced by employing a stochastic method such as MC. The approach itself is based on mean-field (MF) theory, and has been applied previously in various guises to the study of plastic crystals and frustrated magnets (Naya, 1974 ▸; Nagai, 1982 ▸; Descamps, 1982 ▸; Derollez *et al.*, 1990 ▸; Enjalran & Gingras, 2004 ▸; Paddison *et al.*, 2013 ▸; Paddison, 2020 ▸). We anticipate that a suitably generalized methodology could be of enormous value in the investigation of disordered crystals beyond these two specific cases.

Our paper is arranged as follows. We begin by presenting the underlying theory for the MF calculation of single-crystal diffuse scattering from a given interaction model. In Section 3 we briefly introduce the disordered physical system that will form the basis of our proof-of-principle study and develop a related simplified 2D toy model. In both cases we present the corresponding single-crystal diffuse scattering patterns and set as our challenge the task of recovering from this scattering the underlying physics responsible for driving complexity. Section 4 sets out the results of our MF analysis, which we compare against the results of conventional approaches based on reverse Monte Carlo (RMC) and Warren–Cowley (WC) methodologies. We consider in turn both the 2D toy model and its 3D (physical) parent. This section concludes with a discussion of the relative sensitivities of different approaches to data loss; a key result is that the MF approach is remarkably robust in this respect. In the final Section 5 we summarize the opportunities and challenges for generalizing this MF method­ology to enable the systematic investigation of complexity in a wide range of different materials.

## 2. Theory

Mean-field theory is a self-consistent field theory, widely used in statistical physics to model high-dimensional random systems (Curie, 1895 ▸; Weiss, 1907 ▸; Kadanoff, 2009 ▸). Here, we present the formalism as applied to orientationally disordered molecular crystals, but will come to show how this interpretation might equally well be applied to compositional disorder.

### 2.1. Mean-field formalism

Our starting point is to define a suitable pair-interaction Hamiltonian for our particular system of interest. We consider the system as comprised of individual building blocks (*e.g.* molecules), which (i) can adopt any one of a fixed number of discrete orientations, (ii) are positioned on a periodic lattice, and (iii) interact with one another in a pairwise sense. The generalized Hamiltonian is then [following Naya (1974 ▸)]: 

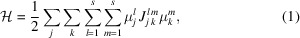

where *j* and *k* sum over all unit cells in the crystal, and *l* and *m* sum over all *s* possible orientations of the disordered mol­ecule. The variables 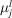 are equal to 1 if the molecule at site *j* is in orientation *l* and 0 otherwise; the 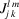 are the components of the pair-interaction Hamiltonian.

It will be convenient for us to express equation (1) in matrix form: 

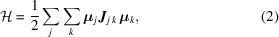

where **μ**_*j*_ is the *s*-dimensional vector with components 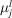 and 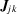 is an *s* × *s*-dimensional interaction matrix.

Working within the same formalism, the diffuse scattering intensity *I*(**q**) is given by 

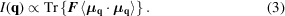

Here, 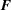 is the *s* × *s*-dimensional matrix of molecular form factors and **μ**_**q**_ is the Fourier transform of **μ**_*j*_. The angle brackets 〈 〉 denote the expectation value.

The MF approximation as taken by Naya (1974 ▸) allows us to express the diffuse scattering intensity in terms of the pair-interaction Hamiltonian,

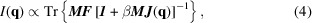

where 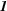 is the identity matrix and 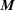 is the *s* × *s* matrix of average orientational populations, with elements 

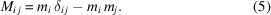

Here, *m*_*i*_ is the probability of finding a molecule in the orientation *i*; *i.e.* the average occupancy of the building block *i*. For the special case where all *s* orientations occur with the same probability 1/*s*, equation (5) reduces to 

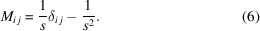




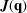
 is the Fourier transform of the pair-interaction matrix and 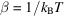 is the inverse thermodynamic temperature, where *k*_B_ is the Boltzmann constant.

This expression can be recast as an eigenvalue problem, 

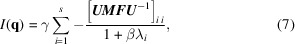

where 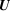 is the matrix that transforms 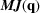 into diagonal form and λ_*i*_ are the eigenvalues of 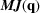 . The parameter γ is a scale factor. Note that the contribution of a given eigenmode to the scattering function is numerically greatest when the corresponding eigenvalue is large and negative.

By assigning a cost function 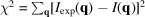 to quantify the difference between experimental and MF diffuse scattering intensities, one can use conventional least-squares approaches to refine the entries in the interaction matrices 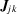 .

### 2.2. Approximations in the MF derivation

The approach used by Naya (1974 ▸) is developed in the random-phase approximation. In this approximation, the 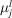 that describe the occupation of the disordered sites in the crystalline system are treated as random variables. The expectation value 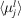 is given by the average occupation of the molecular component as determined by average structure analysis. For the systems presented here, this average occupation is constrained by the average symmetry and is constant for all different orientations.

The local correlations, and the constraint that in the real disordered system each site is occupied by exactly one orientation, collectively imply that the 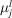 are not independent random variables. In the MF approximation, the central limit theorem is applied and it is assumed that the probability distribution of **μ**_**q**_ is given as an *s*-dimensional Gaussian probability distribution (Naya, 1974 ▸),

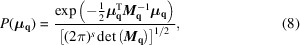

with 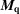 the matrix of the second moments of the random variable **μ**_**q**_.

The random-phase approximation – and hence our MF analysis – is expected to break down for ordered phases, such as an antiferromagnetic Ising system at low temperatures. This is the case when the pair-interaction energies are large with respect to the available thermal energy. Naya (1974 ▸) gives the criterion 

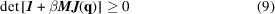

to describe the temperature range in which the MF approximation might be expected to give physical results. In the related MF formalism developed for frustrated magnets (Enjalran & Gingras, 2004 ▸), an equivalent criterion is used that depends on the most negative eigenvalue λ_min_(**q**) in equation (7),

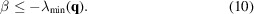

Hence, for all refined pair-interaction energies in the MF approximation presented here, it has to be ascertained that both inequalities (9) and (10) are satisfied.

### 2.3. Number of included pair-interaction terms

In principle, there is no *a priori* limit on the number of pair-interaction terms that might be included in the interaction matrix (2), although our motivation of attempting to describe complex structures succinctly implies that we wish in due course to use as few terms as possible. If too many parameters are used, and their values subsequently refined against experimental diffuse scattering data as outlined above, then such refinements are often unstable. This instability can often be traced back to violation of the criteria in (9) and/or (10).

A suitable strategy, in our view, is as developed by Paddison *et al.* (2013 ▸) for the identification and refinement of magnetic interactions in the frustrated magnet β-Co_*x*_Mn_1−*x*_. Pair-interaction terms are included in a refinement procedure one by one and the goodness of fit monitored. Only terms that lead to a significant improvement can be interpreted in terms of a physical interaction model and should be included in the subsequent analysis.

## 3. Model system

### 3.1. Parent compound

**Figure 2:**
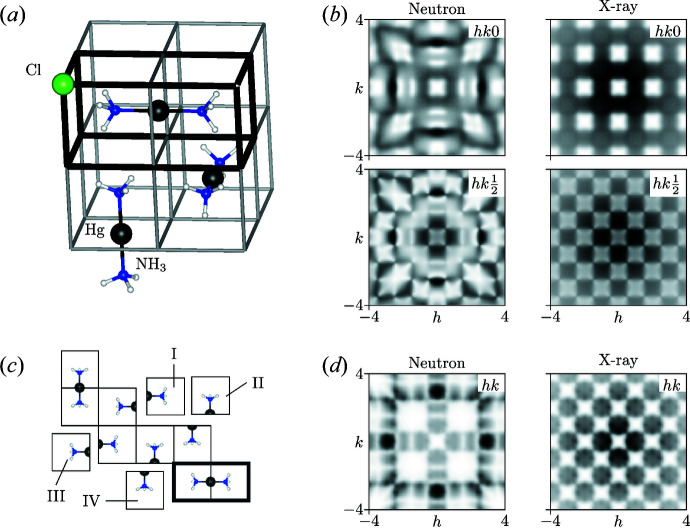
(*a*) A structural model of Hg(NH_3_)_2_Cl_2_, illustrating a possible distribution of Hg^2+^ ions to form [H_3_N–Hg–NH_3_]^2+^ molecules. The Cl^−^ ions occupy the cube vertices (only one such ion is shown). (*b*) Slices of the diffuse neutron and X-ray scattering as calculated from our simulated model structures of Hg(NH_3_)_2_Cl_2_. Note that the only difference between the neutron and X-ray scattering calculations is the difference in scattering factors. (*c*) A simplified 2D structural analogue of Hg(NH_3_)_2_Cl_2_, obtained by projecting onto two spatial dimensions. The four 〈10〉-oriented tiles of [Hg_1/2_–NH_3_]^+^ are labelled and illustrate the matching rules of the system. (*d*) Two-dimensional diffuse neutron and X-ray scattering of the 2D toy model represented in panel (*c*).

For the purposes of this study, we use as our model system the compound Hg(NH_3_)_2_Cl_2_ (Lipscomb, 1953 ▸), chosen because it exhibits strongly correlated disorder that arises from particularly simple local interactions (Parsonage & Staveley, 1978 ▸; Simonov & Goodwin, 2020 ▸) [Fig. 2 ▸(*a*)]. In the physical material, the Cl^−^ ions are positioned on a simple cubic lattice. At the centre of each Cl_8_ cube lies an ammonia molecule, oriented such that its electric dipole points along one of the six 〈100〉 directions. Pairs of NH_3_ molecules in neighbouring cells are connected by Hg^2+^ ions to form [H_3_N–Hg–NH_3_]^2+^ ions that each span a 2 × 1 × 1 ‘brick’ of the cubic Cl^−^ lattice. Hence the orientations of NH_3_ molecules within a single brick are strongly coupled (they point towards one another if connected by a common Hg^2+^ ion), although this local rule is not strong enough to drive long-range orientational order. The system is instead an example of a ‘procrystalline’ material [label *C*_1_ in the notation of Overy *et al.* (2016 ▸)], with 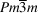 average symmetry.

From a chemical perspective, the structural complexity of Hg(NH_3_)_2_Cl_2_ arises from strongly correlated Hg^2+^ occupancy disorder on the 3*c* Wyckoff position, which couples to orientational disorder on the NH_3_ (1*b*) position. Because we develop our MF formalism in the context of (pure) orientational disorder, we divide the [H_3_N–Hg–NH_3_]^2+^ molecules in two and recast the underlying degrees of freedom in terms of orientations of fictitious (but useful) [Hg_1/2_–NH_3_]^+^ half-molecules. This approach of establishing one-to-one mappings between orientational and compositional disorder problems is well established in the field (Parsonage & Staveley, 1978 ▸; Simonov & Goodwin, 2020 ▸).

### 3.2. Single-crystal diffuse scattering

We generated single-crystal X-ray and neutron diffuse scattering patterns for Hg(NH_3_)_2_Cl_2_ by direct calculation from ensembles of explicit atomistic configurations. The configurations themselves were generated using a ‘loop-move’ MC algorithm (Melko *et al.*, 2001 ▸; Evertz, 2003 ▸), which enabled efficient sampling of the manifold of configurations strictly obeying the [Hg_1/2_–NH_3_]^+^ half-molecule matching rules. A total of 50 ground-state configurations were generated in this way, each corresponding to a 40 × 40 × 40 supercell of the 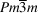 unit cell. Local bond lengths were taken from the related structure of HgNH_2_Cl (Lipscomb, 1953 ▸), and we included rotations of the NH_3_ molecules around the N—Hg bond axis as identified from infrared spectroscopy measurements (Ebisuzaki *et al.*, 1982 ▸). To speed up the calculation, we exploited the fast Fourier calculation algorithm developed by Paddison (2019 ▸). We further improved the statistics of the calculated *I*(**q**) functions by applying 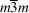 Laue symmetry. Key slices of the diffuse scattering patterns are shown in Fig. 2 ▸(*b*); note that the only difference between the X-ray and neutron scattering is the difference in the scattering factors of the atoms. While the X-ray scattering is dominated by the contributions from Hg, scattering from the lighter N and H atoms dominates the neutron diffraction pattern. The ‘pinch points’ observed in the 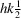 layer arise from the strict local ordering rule. Full details of our calculations and a short discussion on the form of the diffuse scattering are given in the supporting information.

### 3.3. Two-dimensional toy model

It will suit our purposes to establish proof of principle of the MF approach using a simplified model of Hg(NH_3_)_2_Cl_2_, obtained by projecting onto two spatial dimensions. Because the Cl^−^ ions are ordered, we omit them altogether from this model. The six 〈100〉 [Hg_1/2_–NH_3_]^+^ orientations of the parent 3D model are replaced by four 〈10〉-oriented tiles in two dimensions [Fig. 2 ▸(*c*)]; equivalent matching rules apply. Formally, the set of fully matched tilings now corresponds to the *S*_1_ procrystalline model (Overy *et al.*, 2016 ▸) and maps to the ground-state of the ‘square dimer’ model (Kasteleyn, 1961 ▸).

One particular advantage of a 2D model is that the corresponding diffuse scattering pattern is more straightforwardly presented. We show in Fig. 2 ▸(*d*) the X-ray and neutron *I*(**q**) functions calculated for the reciprocal-space region −4 ≤ *h*, *k* ≤ 4. This calculation was again based on loop-move-derived MC configurations (50 multiples of 100 × 100 supercells), but was now carried out using the *DISCUS* program (Neder & Proffen, 2008 ▸). The simulation procedures are described in more detail in the supporting information.

### 3.4. Articulating the challenge to be addressed

Our key goal is to establish whether, having measured the diffuse scattering patterns of the type shown in Figs. 2 ▸(*b*) or 2 ▸(*d*), we can use an MF-based approach to extract the physical interactions responsible for strongly correlated disorder in the relevant 2D or 3D systems – in other words, to recover the underlying matching rules that ultimately provide a succinct interaction-space description of the complex real-space order. This contrasts both atomistic and correlation function approaches, against which we will come to compare our results. We start in 2D, progress to 3D and conclude our results by assessing resilience to data loss.

## 4. Results

### 4.1. Proof-of-concept: 2D toy model

#### 4.1.1. Viability of MF approach

Before attempting an MF-based refinement of diffuse scattering data, we must first establish that the *I*(**q**) function calculated using equation (7) for a sensible interaction model actually provides an accurate representation of the explicit result for our 2D toy model [Fig. 2 ▸(*d*)]. Consequently, we assemble a set of interaction matrices based on penalizing neighbour-tile pairings that violate the matching rules described above. Consider, by way of an example, the forbidden neighbours in the (1, 0) direction for the tile labelled III in Fig. 2 ▸(*c*): each of tiles II, III and IV would leave an un-matched half-Hg and hence we assign a penalty *j* > 0 to these pairings. By contrast, it is only a I tile that is forbidden from neighbouring another I tile in this same orientation. Enumerating all possibilities, we arrive at the interaction matrix 

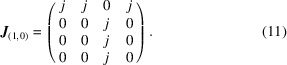

Equivalent matrices for the (−1, 0), (0, 1) and (0, −1) directions are generated straightforwardly by symmetry.

**Figure 3:**
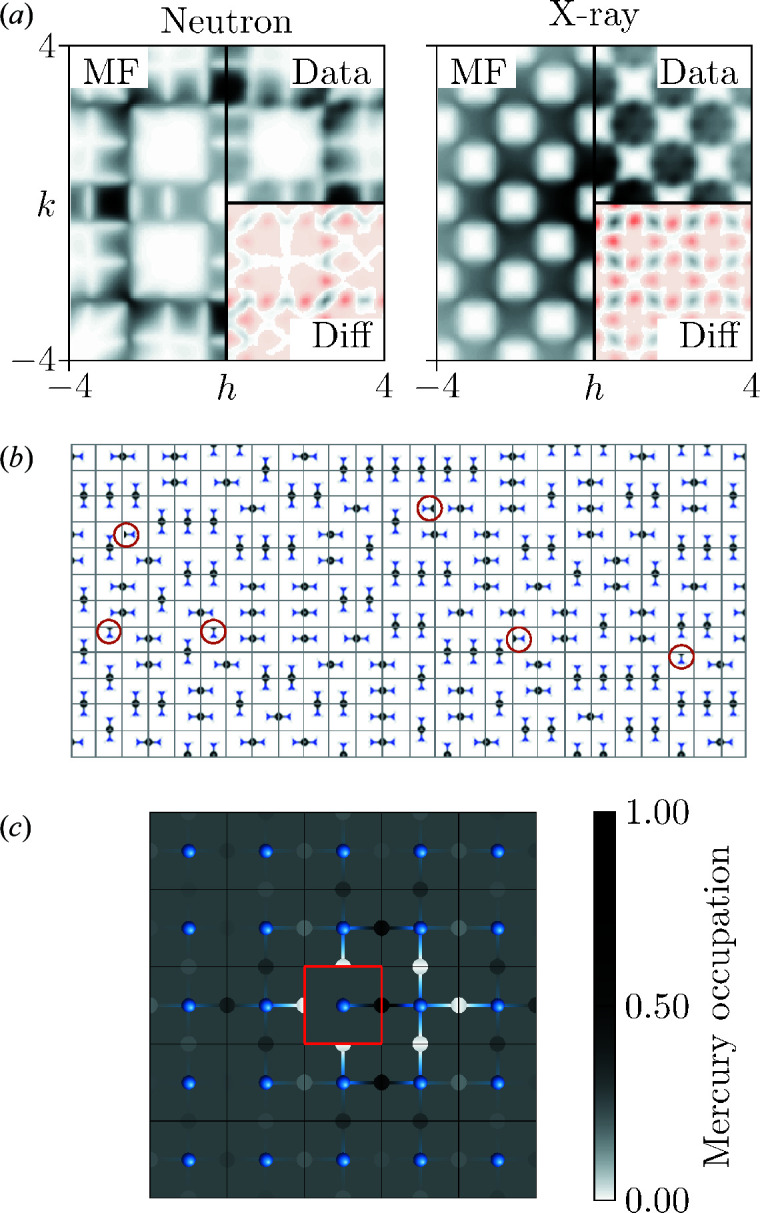
(*a*) Two-dimensional neutron and X-ray diffuse scattering calculated from the MF refinement (left) are compared with the calculated data (top right). The lower right-hand corner shows the difference *I*_MF_ − *I*_exp_, where positive (negative) values are displayed on a white to black (red) colour scale. (*b*) A section of a sample configuration generated by a direct MC simulation driven by the pair-interaction Hamiltonian of equation (11) with β*j* = 1.617. Instances where the tiles are not matched correctly are circled. (*c*) The correlation function of the configuration shown in panel (*b*) averaged for symmetry. A tile orientation III is chosen as a reference, indicated by the red box. The probabilities of the different orientations of the neighbouring tiles are indicated by the occupation probability of the Hg_1/2_ according to the black and white scale shown on the right-hand side. The ammonia groups are indicated in blue for reference. The nearest-neighbour matching rules are fulfilled to a great extent, as can be seen by the white circles, representing forbidden orientations, that surround the red box.

With access to 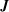 , and using the starting value β*j* = 0, equation (7) can be used to calculate *I*(**q**), and a goodness of fit to the reference data – both X-ray and neutron – can be calculated. The value of β*j* was subsequently refined using least-squares minimization; note that the optimal scale factor γ can be derived analytically at each step of the minimization. All derivatives were calculated numerically and 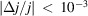 was used as the convergence criterion. The refinement was repeated using all 50 model data sets, allowing us to obtain estimates of the parameter uncertainties; full details of our refinements are given as supporting information. Visual inspection of the calculated diffuse scattering patterns makes clear that the MF approach is indeed capable of capturing its form [Fig. 3 ▸(*a*)], with the biggest deviation arising at the pinch points [see *e.g.* the pinch point at (2.5, 2.5)]. The corresponding *R* values were 13.4 (7) and 14.6 (9)% for, respectively, neutron and X-ray scattering patterns. Both data sets gave very similar values of β*j*, each of which satisfies the MF approximation criterion: 1.750 (4) (neutron) and 1.617 (9) (X-ray). We consider it remarkable that such high-quality fits can be achieved using just one parameter.

Of course, a key advantage of this ‘interaction-space’ solution is that, despite its terseness, it can nonetheless be used to generate a real-space realization of the model of arbitrary physical size. We used MC simulations, driven by the interaction matrices exemplified by equation (11) and the MF value of β*j* = 1.617, to obtain a series of atomistic configurations of the 2D system. A region of one such configuration is illustrated in Fig. 3 ▸(*b*); what is clear is that the matching rules are indeed extremely well obeyed, with very few mismatch defects. In principle, defects are only strictly forbidden in the athermal (β → ∞) limit. Mean-field theory is well known to overestimate ordering temperatures, and so one does not necessarily expect the ‘true’ system and MF temperatures to agree quantitatively, especially in cases with strong correlations (Parisi, 1988 ▸). The direct MC configurations can also be used to calculate the orientational correlation function, shown schematically in Fig. 3 ▸(*c*). Note that non-vanishing correlations are observed for longer-range neighbour pairs beyond those included in the interaction Hamiltonian. We will return to this point in due course.

#### 4.1.2. Model-agnostic MF refinement

In assembling the interaction matrices above [*e.g.* as in equation (11)] we have exploited our *a priori* understanding of the interactions between neighbouring tiles. Consequently, our next step is to ascertain whether the form of the interaction matrix might itself be determined by refinement against diffuse scattering data. It can be shown that there are exactly seven symmetry-inequivalent terms in the nearest-neighbour tile-pair inter­action matrices; the universal form for the (1, 0) direction, by way of example, is 

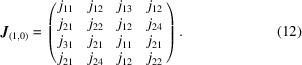

As before, equivalent matrices for the (−1, 0), (0, 1) and (0, −1) directions are obtained by symmetry; no additional *j*_*xy*_ terms are required beyond those in equation (12).

**Figure 4:**
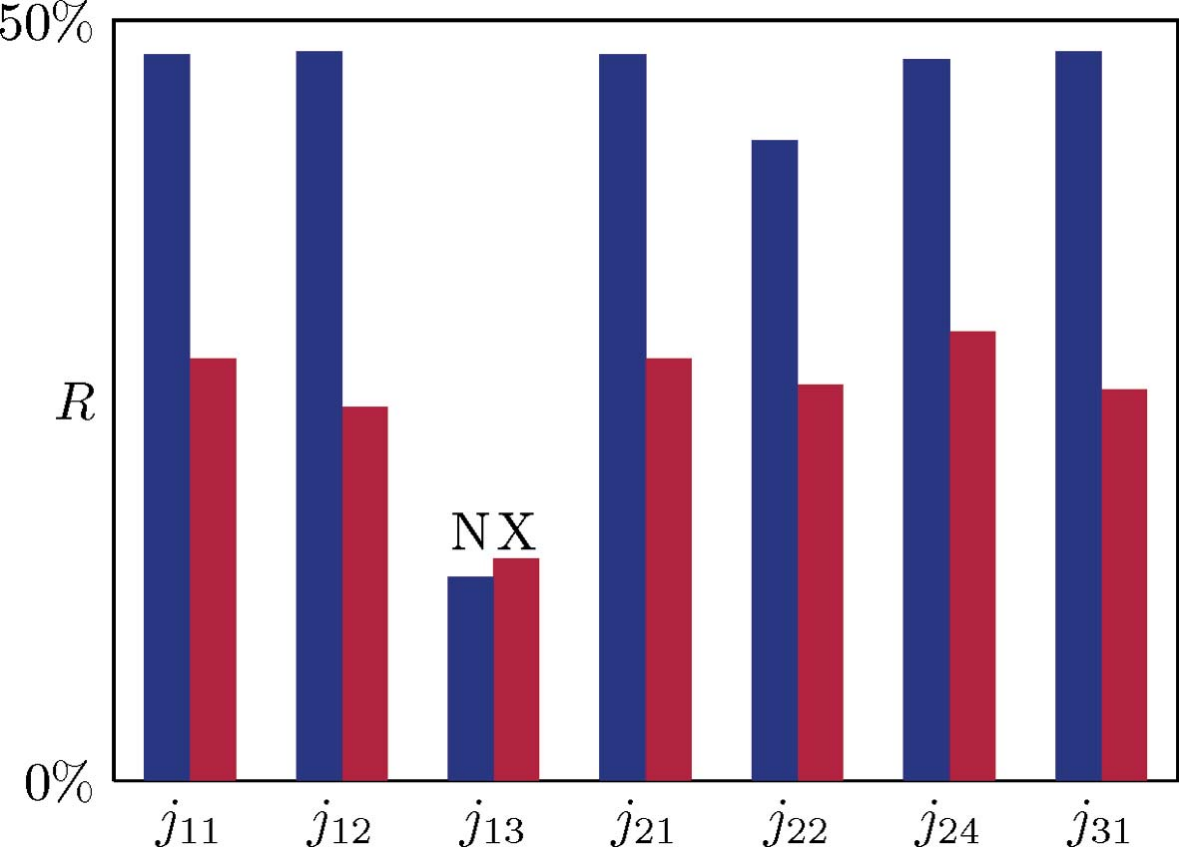
The variation in *R* values for the model-agnostic MF refinement with the different *j*_*xy*_ of equation (12). Neutron scattering (N) values are shown in blue and X-ray scattering (X) in magenta.

We used our model data sets to carry out a series of MF refinements in which we fixed all but one of the *j*_*xy*_ parameters to be zero. The quality of the fit to the neutron and X-ray diffuse scattering patterns as a function of the single *j*_*xy*_ parameter allowed to refine is represented graphically in Fig. 4 ▸. In six of the seven cases, the fits obtained are poor. But in the case where *j*_13_ is allowed to refine then we obtain fits of exactly the same quality as in our test case above – the calculated diffuse scattering patterns in the two instances are indistinguishable (see the supporting information). It can be shown analytically that equations (11) and (12) lead to the same eigenvalue problem when *j*_13_ = −2*j* and *j*_*xy*_ = 0 otherwise. We indeed find the refined values of *j*_13_ to be equal to −2*j*. In retrospect, this equivalence is straightforward to understand: the underlying physics responsible for complexity in this system can be couched either in terms of penalizing forbidden tile pairs (*j* = *j*_11_ = *j*_12_ > 0) or, equivalently, in terms of rewarding matching tile pairs (*j*_13_ < 0) in favour of all other possibilities.

In principle, one might develop the refinement further by allowing one or more *j*_*xy*_ terms, in addition to *j*_13_, to refine, or by including interactions beyond nearest neighbours. In this case, of course, doing so does not improve the fit to the data further than the optimal solution we have already found. As flagged by Paddison *et al.* (2013 ▸), this is the point at which one might claim to have ‘solved’ the problem of identifying the key interactions to which the diffuse scattering data are sensitive.

#### 4.1.3. Comparison with established refinement approaches

By this point, we have shown that the single-crystal diffuse scattering patterns of Fig. 2 ▸(*d*) can be fitted using an MF approach with a single interaction parameter, and that the corresponding interaction model can in turn be used to generate atomistic representations of the underlying dis­ordered structure and also the corresponding correlation functions. We now compare these results with those obtained using conventional refinement strategies that aim to fit the diffuse scattering data directly in terms of atomistic configurations on the one hand and correlation functions on the other.

We used a custom RMC code to refine atomistic configurations against the 2D neutron and X-ray diffuse scattering patterns of Fig. 2 ▸(*d*). Each RMC configuration represented a 20 × 20 supercell (*i.e.* 400 orientational parameters); we used ten independent runs for each of the 50 model data sets and exploited the fast Fourier transform algorithm of Paddison (2019 ▸) in our diffuse scattering calculations. While the quality of the fit to the data is much better than for the MF approach [*R* = 6.6 (1)% (neutron) and *R* = 5.2 (1)% (X-ray)] – perhaps unsurprising given the 400-fold increase in the number of parameters – the fraction of correctly matched tiles is only 75.8 (1)% for the neutron data, and as low as 36.9 (1)% for the X-ray data. This difference in sensitivity is to be expected, since in the neutron case the H atoms and the off-centred N atoms make a significant contribution to the molecular form factor, but in the X-ray case the variations of the molecular form factor in reciprocal space are much weaker because the scattering is dominated by Hg. In either case, the underlying physics responsible for the diffuse scattering is more difficult to extract from these refinements – and arguably impossible from the X-ray scattering alone – than for the MF approach developed above.

**Figure 5:**
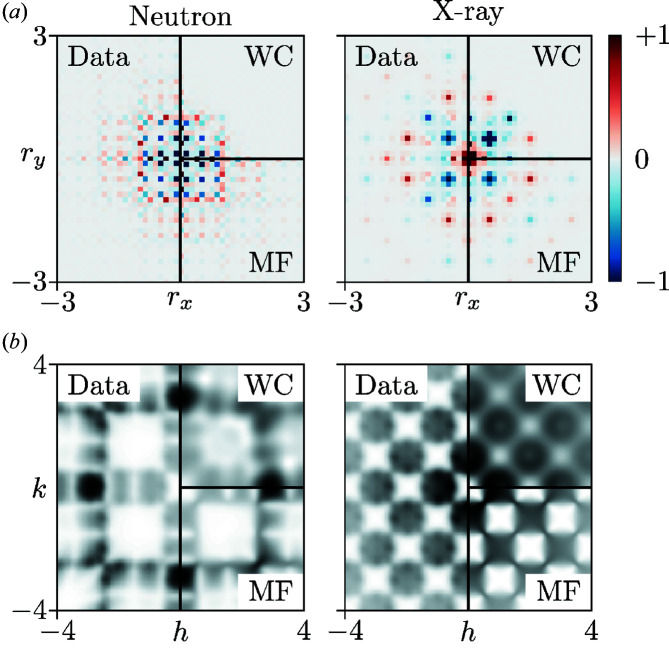
(*a*) A comparison of the 2D-ΔPDFs calculated from the diffuse scattering of the model data (left of panel), the WC refinement (top right of panel) and MF refinement (bottom right of panel). Positive correlations are indicated in red and negative correlations in blue. (*b*) A comparison of the diffuse scattering used to calculate the 2D-ΔPDFs in panel (*a*).

The pairwise correlations from which the diffuse scattering arises can be accessed through the inverse Fourier transform of the diffuse scattering intensities; the corresponding function, known as the 2D-ΔPDF (Simonov *et al.*, 2014*a* ▸), is weighted by the X-ray/neutron atomic scattering factors and is shown in Fig. 5 ▸(*a*) for our toy model. Some direct interpretation of these functions is possible. For example, the negative peak at **r** ∈ 〈½, ½〉 in the X-ray 2D-ΔPDF implies that Hg–Hg contacts at these vectors are forbidden. Quantitative refinement of the 2D-ΔPDF is possible using the *YELL* code (Simonov *et al.*, 2014*b* ▸). We used a suitably customized version (Schmidt & Neder, 2017 ▸) to refine WC correlation parameters for nearest neighbours; by exploiting various symmetry relations there are just three independent parameters to be determined (full details are given in the supporting information). These parameters effectively define the probabilities of different tile pairs, and our results correspond to a better-than-RMC – but not yet perfect – observation of the original matching rules: the rules are obeyed 87.6 (5)% and 76.8 (57)% of the time for correlations extracted from neutron and X-ray data, respectively. In reciprocal space, the corresponding diffuse scattering patterns provide a reasonable match to the data [*R* = 19.9 (2)% (neutron) and *R* = 15.4 (9)% (X-ray)], but are much broadened because they are generated using correlations only at small **r** values [Fig. 5 ▸(*b*)]. A better fit to the data would require additional WC terms to be included in the refinement. Our WC refinements are computationally more expensive than the corresponding MF refinements: both are based on a least-squares algorithm but the WC refinement includes more parameters. A numerical comparison of different refinement times is provided as supporting information.

An important point is made by comparing the 2D-ΔPDFs obtained using our MF approach on the one hand and those represented by this *YELL* (WC) refinement on the other hand. Recall that the former arises from refining nearest-neighbour *interactions* and the latter from nearest-neighbour *correlations*. The MF 2D-ΔPDF is structured beyond the nearest-neighbour positions, because short-range interactions can nonetheless affect longer-range structure [a famous example being the order-by-disorder transition in hard-sphere fluids (Alder & Wainwright, 1957 ▸; Hoover & Ree, 1968 ▸)]. So despite including fewer refinable parameters, the MF approach actually has the potential to give rise to a more detailed structural model. This is the nub of our argument in favour of an ‘interaction-space’ refinement strategy, as exemplified in the MF approach we present here.

One difference in the 2D-ΔPDFs in Fig. 5 ▸(*a*) is at **r** ∈ 〈1, 1〉. Both the MC-generated data and the corresponding WC refinement show a negative correlation at these separations, while the MF refinement predicts a small positive correlation. We tested whether the discrepancy could be accounted for by finite-size effects and found that it cannot (see the supporting information for further discussion). Instead, we suggest that this difference reflects a limitation of the MF approximation for this particular system. Nonetheless, the correct (negative) correlation value is recovered in MC simulations driven by the MF interaction model, and, after all, it is the set of interactions that we seek to extract from the diffuse scattering.

### 4.2. Three dimensions

We turn now to the arguably more physical problem of correlated disorder in the three-dimensional procrystal Hg(NH_3_)_2_Cl_2_. Our approach follows closely that described above for the 2D toy model, and we find ourselves able to draw essentially the same conclusions. We summarize below the key results of MF, RMC and WC refinements against both X-ray and neutron simulated diffuse scattering data. In each case, the square tiles of the 2D model are replaced by cubic ‘blocks’ that correspond to the six possible orientations of [Hg_1/2_–NH_3_]^+^ half-molecules to be arranged within the simple cubic Cl^−^ lattice.

**Figure 6:**
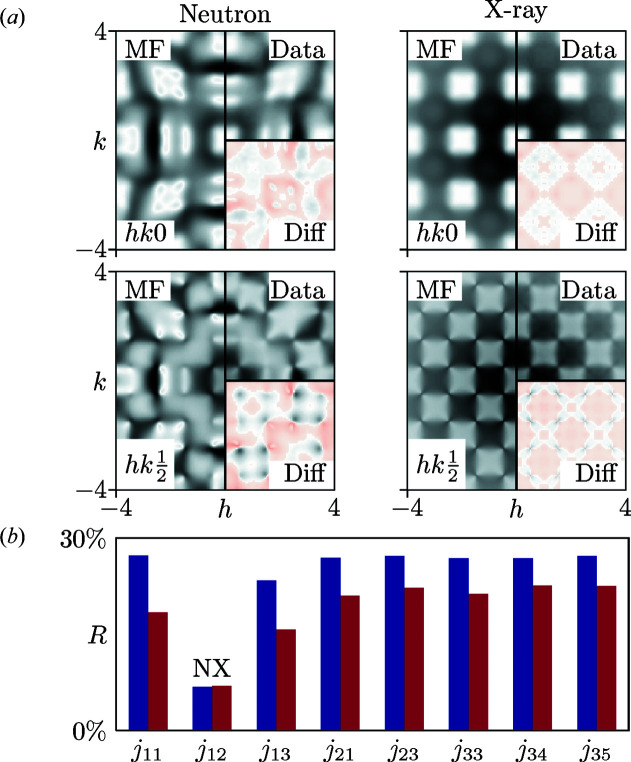
(*a*) Slices of the 3D diffuse neutron and X-ray scattering calculated from our MF refinement (left) compared with the calculated data (top right). The lower right-hand corner shows the difference *I*_MF_ − *I*_exp_, where positive (negative) values are displayed on a white to black (red) colour scale. (*b*) The variation in *R* values for the model-agnostic MF refinement of the 3D data with the different *j*_*xy*_. See the supporting information for further details of the nomenclature and the complete pair-interaction Hamiltonian. Neutron scattering (N) values are shown in blue and X-ray scattering (X) in purple.

The MF equation (7) gives an excellent representation of both neutron and X-ray diffuse scattering patterns for nearest-neighbour interaction matrices that assign a single common energy penalty *j* to mismatched neighbour blocks. The refined values of β*j* = 2.518 (3) (neutron) and 2.959 (10) (X-ray) satisfy the MF approximation criteria, and the remarkable goodness-of-fit values *R* = 6.7 (2) and 4.2 (3)% are even better than for the 2D model. Morever, the data themselves can again be used to determine the form of the interaction matrices without presuming their form. There are now eight independent entries of the interaction matrices, and only one (*j*_12_) is needed to obtain high-quality fits to the data (Fig. 6 ▸). This key parameter encodes the matching rules and can be used to drive MC simulations that obey these rules (see the supporting information for further discussion). So again we conclude that, with a single refined parameter, the MF approach can extract from either neutron or X-ray diffuse scattering data the microscopic mechanism responsible for complexity in this representative system.

RMC refinements are somewhat less successful, and especially so for the case of X-ray diffuse scattering patterns. Our series of ten independent refinements of ten model data sets used 6 × 6 × 6 supercells (216 parameters), yet the fraction of correctly matched tiles was 82.22 (3)% when driven by the neutron diffuse scattering data, and only 21.44 (3)% for the X-ray data. Hence the additional degrees of freedom in RMC *versus* MF approaches serve simply to open up a large configurational space of models that are unphysical yet nonetheless capable of reproducing aspects of the experimental diffuse scattering patterns. The full results of these refinements and the corresponding fits to the data are given as supporting information.

Finally, we also carried out a WC correlation parameter refinement, confining ourselves to nearest-neighbour terms. There are six independent variables involved in these fits, and again full details are provided in the supporting information. For the same reason as discussed in the context of our 2D model, the calculated diffuse scattering is necessarily broadened with respect to experiment, but the matching rules are much more faithfully observed than in the RMC case: the refined correlation parameters correspond to 96 (3)% and 83 (7)% of correctly matched tiles for neutron and X-ray data, respectively.

Across these three different refinement strategies, the best fits to data, the most favourable data-to-parameter ratio and the clearest path from measurement to identifying the underlying physics responsible for complexity are again all given by the MF approach.

### 4.3. Stability against missing data

Since the MF formalism allows such a parameter-efficient means of fitting diffuse scattering data, we sought to establish the extent to which the approach might tolerate incomplete data. We have some experience in this regard from earlier studies of magnetic diffuse scattering, where 2D slices of the full 3D magnetic diffuse scattering pattern have been shown to be sufficient for robust refinement of the corresponding spin interaction model (Paddison *et al.*, 2013 ▸). In the case of conventional (non-magnetic) scattering it is usually possible, if time-consuming, to measure complete single-crystal diffuse scattering patterns, at least under ambient conditions (Welberry & Weber, 2016 ▸). However, the use of sample environments, *e.g.* diamond anvil cells for high-pressure measurements (Katrusiak, 2008 ▸), gas cells (Yufit & Howard, 2005 ▸) or electric field cells (Gorfman *et al.*, 2013 ▸), often imposes severe constraints on reciprocal-space coverage. Likewise, even if the 3D reciprocal space is well covered in a measurement, it can be difficult to determine accurate diffuse scattering intensities close to the Bragg reflections; this is the motivation for so-called ‘punch and fill’ algorithms (Kobas *et al.*, 2005 ▸), which intentionally discard data in these regions of the diffraction pattern. Consequently, the ongoing challenge of linking structural complexity to material function may be aided by the development of efficient refinement strategies that are robust to partial data loss.

We used our 2D toy model and its neutron/X-ray diffuse scattering functions to test the implications of data loss in three cases:

(i) The omission of scattering intensities at and near Bragg reflections, mimicking the ‘punch’ aspect of the punch-and-fill approach;

(ii) The restriction of data to a 10° wedge, such as might be encountered when using a diamond anvil cell; and

(iii) The limiting case of a 1D cut, taken along the (*h*0) reciprocal-space axis.

For each scenario we carried out an MF refinement employing the interaction matrices related to equation (11), and we illustrate here the success of this refinement by attempting to reconstruct the full 2D diffuse scattering pattern. We then compare this reconstruction against those generated using RMC and WC refinements.

**Figure 7:**
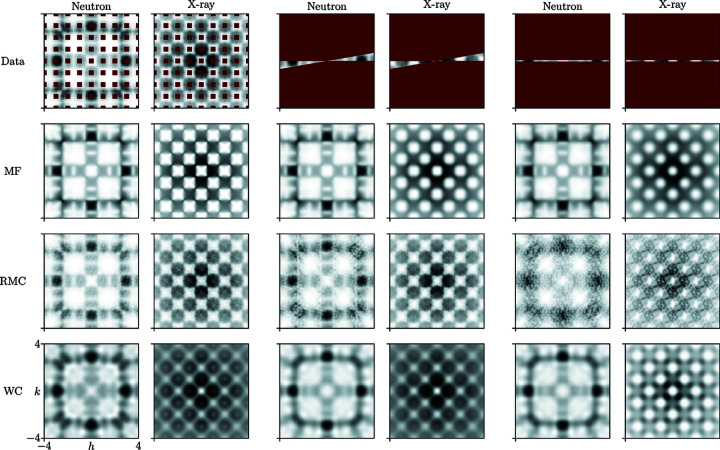
Refinements for missing data input. The top row shows the data input for the three different cases for both neutron and X-ray diffuse scattering: (left) the omission of scattering intensities close to the Bragg reflections, (middle) restriction of the data to a 10° wedge and (right) the 1D *h*0 cut. Omitted data are indicated by magenta blocks. Rows two to four show the complete diffuse scattering as recovered from the refinements to the restricted data input for the MF refinement (row two), RMC refinement (row three) and WC refinement (bottom row).

Our results are summarized in Fig. 7 ▸, with a full analysis and discussion of the extension to three dimensions given in the supporting information. The key observation is that MF refinement is indeed remarkably tolerant of data loss. This is true even in the extreme case of a 1D cut – which omits the key ‘pinch-point’ features – from which the full 2D data can be recovered. Moreover, it is possible to carry out a model-agnostic refinement and identify the correct Hamiltonian (see supporting information). We argue that this robustness arises because the symmetry of the interaction Hamiltonian enforces very strong constraints on the form of the *I*(**q**) function. Symmetry also plays a role in stabilizing the WC refinements, although it is now the (weaker) symmetry of the correlation functions that constrains *I*(**q**). By contrast, RMC is notably intolerant of data loss, which is perhaps unsurprising since it is (by design) agnostic of either crystal or interaction symmetry.

## 5. Concluding remarks and outlook

For the specific procrystalline system Hg(NH_3_)_2_Cl_2_, and its 2D toy analogue, we have established that a mean-field refinement approach allows unambiguous determination from the diffuse scattering patterns of the microscopic interactions that drive their structural complexity. This link can be established without assuming the form of the interactions, and the process is even robust to (extreme) data incompleteness. We cautiously suggest that the interaction-space approach we have taken here might form the basis for a more general strategy for ‘solving’ the structures of complex and/or dis­ordered crystals (Goodwin, 2019 ▸). Certainly, the formalism as presented here is not limited to substitutional disorder in molecular systems with homogeneous average occupancies. The molecular form factors can be easily replaced by atomic form factors, and unequal average occupations are straightforwardly accommodated within the matrix 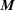 [see equation (5)]. The formalism is easily extended to allow treatment of crystallographic space groups that contain several disordered sites in the unit cell, and such an extension would allow investigation of nonstoichiometric compounds as described by Gusev (2006 ▸) and Withers (2015 ▸).

Looking forward more generally, what challenges might one expect to face? An obvious limitation will be the study of systems poorly described by the mean-field approximation, *e.g.* when the stability criteria are not met. This is the case, for example, for mullite, the diffraction pattern of which contains sharp incommensurate satellite reflections in addition to structured diffuse scattering (Welberry, 2001 ▸). Other cases where our formalism may break down are systems far from equilibrium, or cases that are driven by higher-order inter­actions which cannot easily be reduced to pair interactions (Welberry & Butler, 1994 ▸). Likewise, the formalism as described here relies on discrete degrees of freedom, and so is well suited to problems that can be phrased in terms of occupational disorder, which may include displacements if their magnitude is fixed. The extension to continuous degrees of freedom – needed to capture particular types of displacive disorder, for example – is an important challenge that we are hoping to address in the near future. One expects additional difficulties whenever there is nontrivial interplay between various different degrees of freedom, such as compositional, displacive and magnetic. Nevertheless, we hope to have demonstrated here that the potential reward for developing a generalized mean-field approach to fitting diffuse scattering data may be great indeed.

## 6. Related literature

For further literature related to the supporting information, see Withers *et al.* (2004 ▸).

## Supplementary Material

Additional discussion, figures and tables. DOI: 10.1107/S2052252521009982/ro5031sup1.pdf
