# Supplementary material for: Efficient fitting of single-crystal diffuse scattering in interaction space: a mean-field approach
Source: IUCrJ. 2021 Nov 3;9(Pt 1):21–30. doi: 10.1107/S2052252521009982 (PMC8733889; doi:10.1107/S2052252521009982)
Supplement: Supplementary file 1 [file m-09-00021-sup1.pdf]

IUCrJ

Volume 9 (2022)

Supporting information for article:

Efficient fitting of single-crystal diffuse scattering in interaction space: a mean-field approach

Ella M. Schmidt, Johnathan M. Bulled and Andrew L. Goodwin

Contents

1 Generation of model data **S3**

2D System . . . S3

3D System . . . S3

2 2D Model System Refinement Details **S7**

Details of the mean-field refinement . . . S7

Model-agnostic mean-field refinement . . . S7

Pair correlations for Warren–Cowley analysis . . . S9

Pair correlations for the RMC analysis . . . S10

Refinement times . . . S12

Possible finite size effects . . . S12

3 3D Model System **S14**

Pair-interaction Hamiltonian . . . S14

Model-agnostic refinement . . . S14

DMC simulation driven with the mean-field parameter . . . S16

Warren–Cowley refinement . . . S16

RMC analysis . . . S16

Refinement times . . . S20

4 Stability against missing data **S21**

Supplementary data for the 2D model system . . . S21

Model-agnostic refinement for 2D missing data . . . S23

Missing data in 3D refinements . . . S24

5 References **S25**

1 Generation of model data

2D System

To generate the two-dimensional model systems, in a first step a direct Monte Carlo (DMC) simulation is run on a grid of 100×100 unit cells. The simulation introduces an energy penalty for neighbouring tiles that do not form $[Hg(NH_3)_2]^{2+}$ molecules. The simulation uses periodic boundary conditions and has a zero-energy exit criterion; *i.e.* it creates a structure with no unmatched half-Hg atoms. From this starting structure, the 50 model structures described in the text were then created using so-called 'loop moves', carried out as follows. First, the orientation of one tile is flipped at random. Then the neighbouring tile is flipped to 'repair' the broken $[Hg(NH_3)_2]^{2+}$ molecule, leaving a new unpaired tile. This new unpaired tile is then again flipped at random and repaired with the respective neighbouring tile. This continues until the unpaired tile from the first flip is used to repair a $[Hg(NH_3)_2]^{2+}$ molecule. The sample structures were created from the initial structure by performing between 500 and 1000 consecutive random loop moves. It is worth noting that the fractional populations of the various tile types were not constrained to be exactly 0.25. In the simulated structures the compositions of these different components vary slightly around the average value of 0.25.

The diffuse scattering in the hk-layer ($-4 \leq h, k \leq 4$ and 201×201 pixels) was calculated using the DISCUS program.[S1] Here, the diffuse scattering is calculated using lots of 4×4 unit cells and all lots of the generated sample structures were used in the calculation of the diffuse scattering. Hence, the analysis performed will not account for correlations beyond the fourth neighbour, which is justified considering the interaction model used in the model structure generation and the analysis methods we apply to the model system. But at the same time note, that this finite lot size effectively widens the observed pinch points, to a degree that is negligible in the analysis we perform here.

3D System

As for the two-dimensional model system, each unit-cell contains one $[Hg_{1/2}–NH_3]^+$ half-molecule. To generate the model systems in a first step a DMC simulation is run on a $10 \times 10 \times 10$ unit cell simulation box, that introduces an energy penalty for neighbouring half Hg atoms that are not paired correctly. The simulation uses periodic boundary conditions and has a zero-energy

exit criterion; *i.e.* it creates a structure with no unmatched half-Hg atoms. From this structure, a bigger $40 \times 40 \times 40$ unit cell structure is then created by aligning $4 \times 4 \times 4$ of the smaller model structures. 50 model structures are then created using the three dimensional equivalent of the 'loop moves' discussed above for the two-dimensional system. The sample structures were created from the initial structure by performing between 5 000 and 10 000 consecutive random loop moves.

The diffuse scattering is calculated using a fast Fourier transform for subsitutional disorder as described in Ref. S 2. Instead of atomic form factors the analogue complex molecular form factors for each of the six orientations were used. The molecular form factors were orientationally averaged for the rotation of the NH_3 group around the Hg–N bond. For the resampling of the diffuse intensity in reciprocal space the Lanczos resampling approach is taken and the parameters $m = 2$ and $m' = 6$ are chosen. Therefore correlations that are beyond the second shell of neighbours are suppressed in the calculation which is justified given the used interaction model. The diffuse scattering was calculated for a volume defined by $-4 \leq h, k, l \leq 4$ on a grid of $161 \times 161 \times 161$ voxels.

The diffuse X-ray scattering that we observe in Figure 2(b) of the main text for the $hk\frac{1}{2}$-layer and the 2D toy model in the hk-layer strongly resembles the diffuse scattering in the $hk0$ layer observed for the procrystalline solid 'D3' in the notation of Overy *et al.* in Ref. S 3 and the simulation of pure occupational order in BZN ($(Bi_{3/2}Zn_{1/2})(Zn_{1/2}Nb_{3/2})O_7$) simulated by Withers *et al.* in Ref. S 4. In fact, the diffraction patterns are related to each other by a 45° rotation. BZN shows the conventional cubic, $A_2B_2O_6O'$ pyrochlore structure, where the $O'A_2$ lattice forms a network of corner connected tetrahedra. Each of these tetrahedra contains three Bi and one Zn atom, creating the strict local ordering rule of the procrystalline solid 'D3'. The $hk0$ plane of diffuse scattering of these systems corresponds to a projection of the structure along the z-axis which is shown on the left of Figure S1. The local ordering rule then implies that two neighbouring tetrahedra are connected by sharing a Bi atom (one possible ordering scheme is indicated by the red lines). This ordering scheme is identical to the ordering scheme in our two-dimensional structural analogue (except for small form factor effects), if rotated by 45° as shown on the right of Figure S1. Hence, we expect the diffuse scattering to show identical patterns, related by the same 45° rotation.

We observe the same diffuse scattering in the $hk\frac{1}{2}$-layer of our three-dimensional parent

Figure S1: Projection of the O′A_2 tetrahedra of the pyrochlore structure along the z-axis (left) compared to our two-dimensional structural anaogue (right). The red line correspond to one realization of the strict local ordering rule that connects neighbouring tetrahera by sharing one Bi atom or connects the half Hg atoms in our toy model.

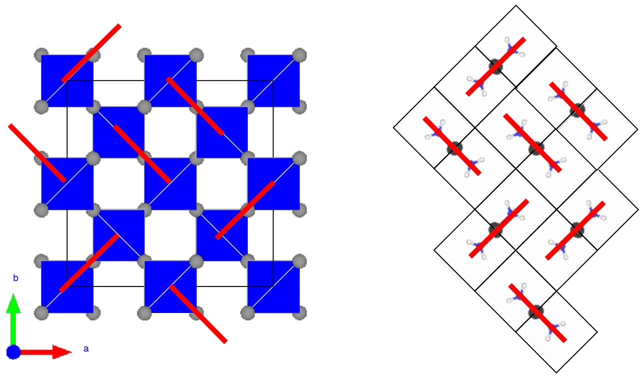

structure. The $hk0$ layer corresponds to a projection of the structure along the z-axis. For our system those $[Hg_{1/2}–NH_3]^+$ with their axis along z are projected onto a single tile, which essentially corresponds to adding an additional tile to our 2D model consisting of a single atom in the centre.

The diffuse scattering in our model system is driven by the forbidden local $\langle\frac{1}{2},\frac{1}{2},0\rangle$ distances. For X-ray scattering the Hg occupation dominates the diffuse scattering and the uniform diffuse Laue scattering is modulated by a function that is proportional to

$$\cos(\pi(h+k)) + \cos(\pi(h-k)) + \cos(\pi(h+l)) + \cos(\pi(h-l)) + \cos(\pi(k+l)) + \cos(\pi(k-l)), \quad (1)$$

where the argument of the cosine function contains the product of the reciprocal space vector $\boldsymbol{h}$ and the forbidden Hg-Hg vectors. This expression can be transformed to give

$$2\cos(\pi h)\cos(\pi k) + 2\cos(\pi h)\cos(\pi l) + 2\cos(\pi k)\cos(\pi l). \quad (2)$$

For the case that $l = \frac{1}{2}$, we have $\cos(\pi l) = 0$ and the expression reduces to

$$2\cos(\pi h)\cos(\pi k), \quad (3)$$

which is identical to the function that modulated the diffuse Laue-scattering of our two-dimensional model system, where $\langle\frac{1}{2},\frac{1}{2}\rangle$ Hg-Hg vectors are forbidden. Hence, the disorder diffuse

X-ray scattering in the $hk\frac{1}{2}$-layer of the three-dimensional parent structure and the hk-layer of the two-dimensional toy-model are generated by the same modulation of the diffuse Laue intensity. Therefore, the $hk\frac{1}{2}$-layer of the three-dimensional parent structure also resembles the simulation of pure occupational order in BZN as simulated by Withers *et al.* in Ref. S4.

2 2D Model System Refinement Details

Details of the mean-field refinement

The pair-interaction Hamiltonian of Equation (9) of the main text has to be Fourier transformed for the mean-field approach:

$$\underline{\underline{J}}_{(1,0)}(h,k) = j \begin{pmatrix} \mathrm{e}^{+2\pi \mathrm{i}h} & \mathrm{e}^{+2\pi \mathrm{i}h} & 0 & \mathrm{e}^{+2\pi \mathrm{i}h} \\ 0 & 0 & \mathrm{e}^{+2\pi \mathrm{i}h} & 0 \\ 0 & 0 & \mathrm{e}^{+2\pi \mathrm{i}h} & 0 \\ 0 & 0 & \mathrm{e}^{+2\pi \mathrm{i}h} & 0 \end{pmatrix}. \tag{4}$$

The pair-interaction Hamiltonians for the $(-1,0)$, $(0,1)$ and $(0,-1)$ directions result from symmetry considerations. This results in the complete Fourier transformed pair-interaction Hamiltonian $\underline{\underline{J}}(h,k)$:

$$\underline{\underline{J}}(h,k) = j \begin{pmatrix} \mathrm{e}^{+2\pi \mathrm{i}h} + \mathrm{e}^{-2\pi \mathrm{i}h} & \mathrm{e}^{+2\pi \mathrm{i}h} + \mathrm{e}^{-2\pi \mathrm{i}k} & 0 & \mathrm{e}^{+2\pi \mathrm{i}h} + \mathrm{e}^{+2\pi \mathrm{i}k} \\ \mathrm{e}^{-2\pi \mathrm{i}h} + \mathrm{e}^{+2\pi \mathrm{i}k} & \mathrm{e}^{+2\pi \mathrm{i}k} + \mathrm{e}^{-2\pi \mathrm{i}k} & \mathrm{e}^{+2\pi \mathrm{i}h} + \mathrm{e}^{+2\pi \mathrm{i}k} & 0 \\ 0 & \mathrm{e}^{-\mathrm{i}2\pi h} + \mathrm{e}^{-2\pi \mathrm{i}k} & \mathrm{e}^{+\mathrm{i}2\pi h} + \mathrm{e}^{-2\pi \mathrm{i}h} & \mathrm{e}^{-\mathrm{i}2\pi h} + \mathrm{e}^{+2\pi \mathrm{i}k} \\ \mathrm{e}^{-\mathrm{i}2\pi h} + \mathrm{e}^{-2\pi \mathrm{i}k} & 0 & \mathrm{e}^{+\mathrm{i}2\pi h} + \mathrm{e}^{-2\pi \mathrm{i}k} & \mathrm{e}^{+\mathrm{i}2\pi k} + \mathrm{e}^{-2\pi \mathrm{i}k} \end{pmatrix}. \tag{5}$$

The refinement algorithm uses Equation (4) of the main text to calculate the diffuse scattering intensity $I(h,k)$ for a given j and the interaction matrix as stated in Equation (2). The derivatives that are involved in the least-squares refinement were calculated numerically by increasing j by 1%. The scale factor γ can be analytically calculated as:

$$\gamma = \frac{\sum_{h,k} I_{\mathrm{exp}}(h,k) \times I(h,k)}{\sum_{h,k} I(h,k)^2}. \tag{6}$$

Model-agnostic mean-field refinement

The average symmetry of the system constrains the pair interactions: symmetry equivalent configurations should yield the same energy penalty or gain. For the $(1,0)$-neighbours the following relations can be derived:

$$j_{11} = j_{33}, \quad (7)$$

$$j_{21} = j_{32} = j_{34} = j_{41}, \quad (8)$$

$$j_{12} = j_{14} = j_{23} = j_{43}, \quad (9)$$

$$j_{24} = j_{42}, \quad (10)$$

$$j_{22} = j_{44}. \quad (11)$$

Figure S2: Neutron and X-ray diffuse scattering as calculated from the mean-field refinement with j_{13} only is identical the the refinement shown in Figure 3 of the main text.

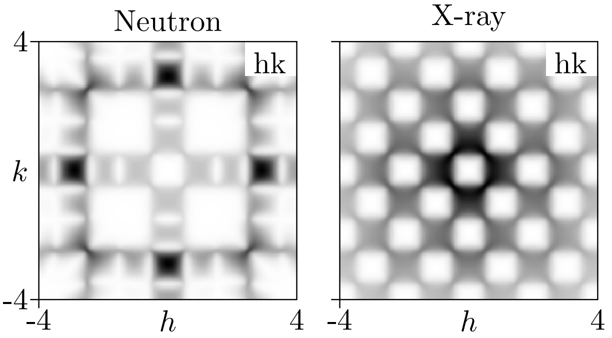

Table S1: Model-agnostic refinement of the mean-field model for the seven different symmetry-independent j_{xy}, as compared to that of j of Equation (9) of the main text.

	j_{11}	j_{12}	j_{13}	j_{21}	j_{22}	j_{24}	j_{31}	j
				Neutron				
R (%)	47.8(2)	47.9(0)	13.4(7)	47.5(2)	42.1(1)	47.4(3)	47.9(3)	13.4(7)
βj_{xx}	−0.48(7)	0.00(0)	−3.50(1)	0.33(3)	−0.98(4)	−0.50(3)	−0.09(6)	1.75(0)
γ	1.28(0)	0.98(0)	0.29(0)	1.27(0)	1.19(0)	1.24(0)	1.29(0)	0.29(5)
				X-ray				
R (%)	27.2(5)	24.5(5)	14.6(1)	30.4(5)	26.0(5)	29.5(3)	25.7(8)	14.6(9)
βj_{xx}	−0.39(2)	0.39(1)	−3.23(2)	−0.16(5)	−0.57(3)	0.48(5)	1.09(5)	1.62(1)
γ	1.90(0)	1.59(0)	0.42(1)	1.79(0)	1.74(0)	1.85(0)	1.75(0)	0.43(1)

These equivalences result in the universal form of $\underline{\underline{J}}_{(1,0)}$ as given in Equation 10 of the main text. The full parameters for each of the refinements are listed in Table S1. The neutron and X-ray diffuse scattering of the refinement with j_{13} only are shown in Figure S2.

The eigenvalues of Equation (5) of the main text are what drives the modulation in the diffuse scattering. They can be calculated analytically for the different cases shown here. This will be used here to illustrate that in fact the Hamiltonian of Equation (9) of the main text and the refinement with j_{13} are equivalent. To substantiate this point we note first that the eigenvalues for $\underline{\underline{MJ}}(h,k)$ for Equation (9) of the main text are given as

$$0, -\frac{j}{2}, \frac{j}{2}, \frac{j}{8}\left[\exp(-2\pi \mathrm{i}h) + \exp(2\pi \mathrm{i}h) + \exp(-2\pi \mathrm{i}k) + \exp(2\pi \mathrm{i}k)\right]. \tag{12}$$

Similarly, the eigenvalues for the refinement with j_{13} are given as

$$0, \frac{j_{13}}{4}, -\frac{j_{13}}{4}, -\frac{j_{13}}{16}\left[\exp(2\pi \mathrm{i}(2h+k)) + \exp(2\pi \mathrm{i}(h+2k)) + \exp(2\pi \mathrm{i}(3h+2k)) + \exp(2\pi \mathrm{i}(2h+3k))\right]. \tag{13}$$

Hence the first three eigenvalues are identical for $j_{13} = -2j$ and have identical eigenvectors, while for the fourth eigenvalue that differs, the difference in the associated eigenvectors yields an identical expression for Equation (5) of the main text. These equations also establish the stability criterion in accordance with Equations (7) and (8) of the main text as

$$-2 \leq \beta j \leq 2 \tag{14}$$

and

$$-4 \leq \beta j_{13} \leq 4, \tag{15}$$

which are fulfilled by the refinements listed in Table S1.

Pair correlations for Warren–Cowley analysis

The Warren–Cowley refinement uses the first neighbour pair correlations only. The average symmetry of the system constrains the pair correlations for the $(1,0)$-neighbours in an identical way to the relations established for the pair-interaction parameters in Equations (4)-(8). With the constraint of the average structure—that each 'tile' is present in 25% of cases—this leads to four symmetry-inequivalent pair-correlations that need to be refined. The refinements were performed using the build-in *fminsearch* function in Matlab, introducing a penalty for pair-correlations that are not in the range between 0 and 0.25. The refinement results are given

in Table S2 along with the $III \rightarrow I$ probability that determines the percentage of correctly matched bonds.

Table S2: Warren–Cowley-refined $(1, 0)$ first neighbour pair correlations for X-ray and neutron scattering in the two-dimensional model system. A pair-probability of 6.25% corresponds to random disorder, while 25% corresponds to perfect order.

pair	Neutron	X-ray
$I \rightarrow I$	2.56(5)%	5.6(9)%
$I \rightarrow II$	6.66(12)%	7.9(4)%
$II \rightarrow I$	2.67(6)%	0.1(3)%
$II \rightarrow II$	16.6(3)%	15.4(16)%
$III \rightarrow I$	21.90(12)%	19.2(14)%

Pair correlations for the RMC analysis

The symmetry-averaged pair probabilities from the RMC analysis are listed in Table S3 and represented graphically in Figure S3.

Figure S3: Symmetry-averaged correlations of the RMC analysis. The occupation of the mercury is depicted indicated by the grey scale shown on the right. (a) Neutron. (b) X-ray.

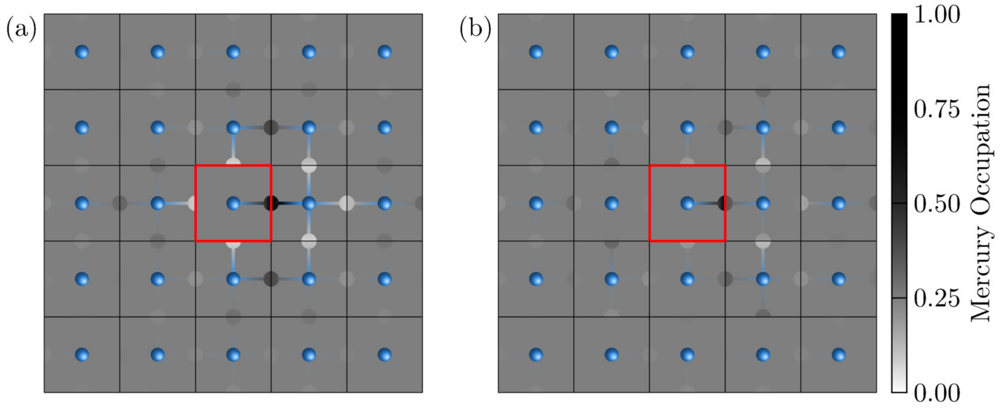

Table S3: Symmetry-averaged pair probabilities from the RMC analysis for the two-dimensional model system. A pair probability of 0.25 corresponds to a completely uncorrelated pair.

	Neutron				X-ray			
	I→I	I→II	I→III	I→IV	I→I	I→II	I→III	I→IV
$\bar{2}\bar{2}$	0.256(2)	0.245(1)	0.244(1)	0.256(1)	0.255(2)	0.248(2)	0.240(0)	0.257(2)
$\bar{2}\bar{1}$	0.260(2)	0.278(1)	0.218(1)	0.244(1)	0.268(2)	0.262(1)	0.215(0)	0.255(2)
$\bar{2}0$	0.308(2)	0.285(1)	0.121(1)	0.286(1)	0.305(1)	0.264(1)	0.166(1)	0.264(2)
$\bar{2}1$	0.260(2)	0.244(1)	0.218(2)	0.278(1)	0.268(2)	0.256(2)	0.214(1)	0.262(1)
$\bar{2}2$	0.255(1)	0.256(1)	0.244(0)	0.245(1)	0.255(1)	0.256(1)	0.241(0)	0.248(2)
$\bar{1}\bar{2}$	0.242(1)	0.278(1)	0.242(0)	0.238(0)	0.239(1)	0.262(1)	0.257(0)	0.242(2)
$\bar{1}\bar{1}$	0.211(1)	0.108(1)	0.397(0)	0.285(2)	0.219(1)	0.125(1)	0.343(1)	0.313(1)
$\bar{1}0$	0.089(1)	0.076(0)	0.758(1)	0.077(0)	0.249(2)	0.193(1)	0.369(1)	0.190(1)
$\bar{1}1$	0.210(2)	0.285(1)	0.397(0)	0.108(1)	0.219(1)	0.314(1)	0.343(2)	0.125(1)
$\bar{1}2$	0.242(1)	0.238(2)	0.243(1)	0.278(1)	0.238(1)	0.241(2)	0.258(1)	0.262(1)
$0\bar{2}$	0.244(1)	0.286(1)	0.237(1)	0.234(1)	0.256(0)	0.264(2)	0.243(3)	0.237(2)
$0\bar{1}$	0.417(1)	0.077(0)	0.212(2)	0.295(0)	0.299(1)	0.190(1)	0.251(1)	0.261(1)
00	1.000(0)	0.000(0)	0.000(0)	0.000(0)	1.000(0)	0.000(0)	0.000(0)	0.000(0)
01	0.417(1)	0.295(1)	0.212(1)	0.076(0)	0.299(1)	0.261(1)	0.248(0)	0.193(1)
02	0.244(1)	0.234(0)	0.237(1)	0.285(1)	0.256(0)	0.237(1)	0.243(1)	0.264(1)
$1\bar{2}$	0.242(1)	0.244(1)	0.258(1)	0.257(1)	0.238(1)	0.255(2)	0.255(1)	0.252(1)
$1\bar{1}$	0.210(2)	0.285(2)	0.261(1)	0.244(2)	0.219(1)	0.313(1)	0.247(0)	0.221(2)
10	0.089(1)	0.295(0)	0.321(2)	0.295(1)	0.249(2)	0.261(1)	0.229(2)	0.261(1)
11	0.211(1)	0.244(2)	0.261(1)	0.285(1)	0.219(1)	0.221(2)	0.246(0)	0.314(1)
12	0.242(1)	0.257(1)	0.258(1)	0.244(1)	0.239(1)	0.251(2)	0.254(1)	0.256(2)
$2\bar{2}$	0.255(1)	0.256(1)	0.244(1)	0.245(0)	0.255(1)	0.257(2)	0.245(0)	0.243(1)
$2\bar{2}$	0.260(2)	0.238(0)	0.245(0)	0.257(1)	0.268(2)	0.242(2)	0.238(1)	0.251(2)
20	0.308(2)	0.234(1)	0.223(0)	0.234(0)	0.305(1)	0.237(2)	0.221(0)	0.237(1)
21	0.260(2)	0.257(1)	0.246(1)	0.238(2)	0.268(2)	0.252(1)	0.238(0)	0.241(2)
22	0.256(2)	0.245(0)	0.244(1)	0.256(1)	0.255(2)	0.243(1)	0.245(1)	0.256(1)

Refinement times

The mean-field refinement and the Warren–Cowley refinement both use a least squares algorithm and hence the total refinement times, which include necessary setup calculations, as well as the calculation times per refinement cycle can be compared (see Table S4). Due to the short observed refinement times our mean-field code was not heavily optimized for computation times. For the RMC refinement, the observed refinement times were larger and the code was optimized for faster calculations. In Table S4 we report the refinement time per RMC move as compared to refinement time per cycle for the two other approaches. In all cases the refinement time was shortest for the mean-field refinement.

Possible finite size effects

In two dimensional systems finite size effects can be significant even for ostensibly large supercells. In our model analysis we used 100×100 unit cells. To exclude the possibility of finite size effects affecting the observed interactions and correlations in our analysis, we simulated one system of 400×400 unit cells and a further system of 1000×1000 unit cells. For these two different configuration sizes, we generated starting configurations by aligning either 4×4 or 10×10 copies of our smaller systems, and then introduced additional disorder by running a large number of loop moves on the resulting configuration (several times the number of tiles per system). The resulting 2D diffuse X-ray scattering and 2D-ΔPDF are shown in Figure S4 and do not show any significant differences. From this observation we conclude that the correlations we observe in our model data are not affected by possible finite size effects.

Table S4: Total refinement times, including setup calculations, and refinement times per cycle for Warren–Cowley and mean filed refinement, and the refinement time per move for the RMC refinement for the 2D toy model system.

	mean-field		Warren–Cowley		RMC	
	Neutron	X-ray	Neutron	X-ray	Neutron	X-ray
Total time (s)	0.35	13.89	12.39	15.48	27.17	27.79
Time per cycle/move (s)	0.09	0.09	0.06	0.06	0.005	0.005

Figure S4: Evaluation of possible finite size effects for the two dimensional toy system. The diffuse X-ray scattering (top row) and the resulting 2D-ΔPDFs (bottom row) are compared to each other for a system size of 100×100 unit cells (left), 400×400 unit cells (middle) and 1000×1000 unit cells (right).

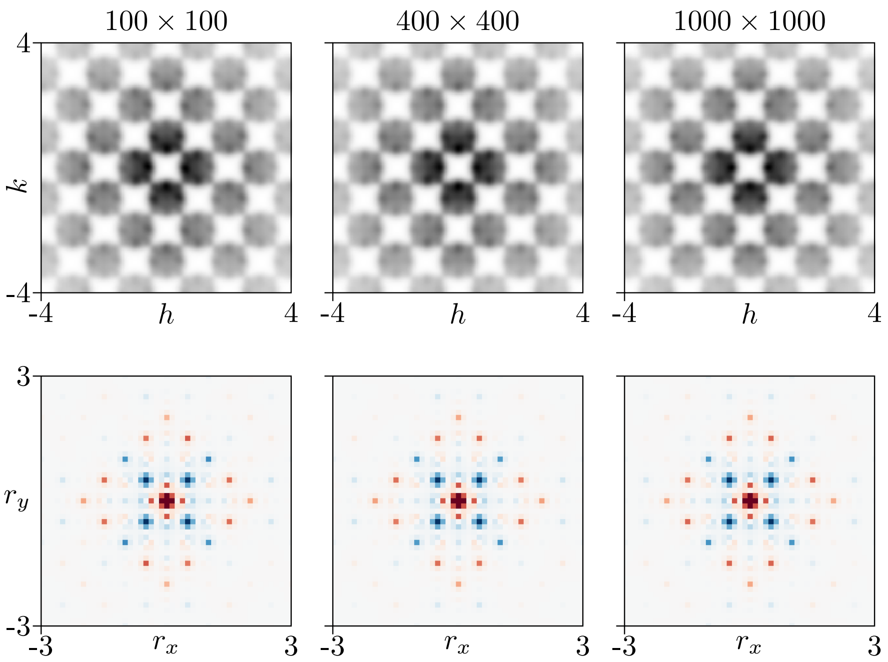

3 3D Model System

Pair-interaction Hamiltonian

In the three-dimensional system, there are six possible orientations of the $[Hg_{1/2}(NH_3)]^+$ half-molecule. We denote as orientation I the half Hg at $(1, 0.5, 0.5)$, orientation II the half Hg at $(0, 0.5, 0.5)$, orientation III the half Hg at $(0.5, 1, 0.5)$, orientation IV the half Hg at $(0.5, 0, 0.5)$, orientation V the half Hg at $(0.5, 0.5, 1)$ and orientation VI the half Hg at $(0.5, 0.5, 0)$. As for Equation (8) in the main text this results in the pair-interaction Hamiltonian for the (1,0,0) direction:

$$\underline{\underline{J}}_{(1,0,0)} = \begin{pmatrix} j & 0 & j & j & j & j \\ 0 & j & 0 & 0 & 0 & 0 \\ 0 & j & 0 & 0 & 0 & 0 \\ 0 & j & 0 & 0 & 0 & 0 \\ 0 & j & 0 & 0 & 0 & 0 \\ 0 & j & 0 & 0 & 0 & 0 \end{pmatrix}. \tag{16}$$

The corresponding pair-interaction Hamiltonians for the $(-1, 0, 0)$, $(0, \pm1, 0)$ and $(0, 0, \pm1)$ directions are related by symmetry. Evaluating the mean-field stability criteria of Equation (8) and (9) of the main text yields

$$-3 \leq \beta j \leq 3, \tag{17}$$

which is fulfilled by the parameters in our refinement (2.518(3) (neutron) and 2.959(10) (X-ray)).

Model-agnostic refinement

For the model-agnostic refinement we need to consider which elements of the pair-interaction Hamiltonian are symmetry equivalent. From symmetry and the nomenclature established in the previous paragraph we derive:

$$j_{11} = j_{22} \tag{18}$$

$$j_{33} = j_{44} = j_{55} = j_{66} \tag{19}$$

$$j_{13} = j_{14} = j_{15} = j_{16} = j_{32} = j_{42} = j_{52} = j_{62} \tag{20}$$

$$j_{23} = j_{24} = j_{25} = j_{26} = j_{31} = j_{41} = j_{51} = j_{61} \tag{21}$$

$$j_{34} = j_{43} = j_{56} = j_{65} \tag{22}$$

$$j_{35} = j_{36} = j_{45} = j_{46} = j_{53} = j_{54} = j_{63} = j_{64}. \tag{23}$$

These equivalences bring us to the pair-interaction Hamiltonian in the most general form:

$$\underline{\underline{J}}_{(1,0,0)} = \begin{pmatrix} j_{11} & j_{12} & j_{13} & j_{13} & j_{13} & j_{13} \\ j_{21} & j_{11} & j_{23} & j_{23} & j_{23} & j_{23} \\ j_{23} & j_{13} & j_{33} & j_{34} & j_{35} & j_{35} \\ j_{23} & j_{13} & j_{34} & j_{33} & j_{35} & j_{35} \\ j_{23} & j_{13} & j_{35} & j_{35} & j_{33} & j_{34} \\ j_{23} & j_{13} & j_{35} & j_{35} & j_{34} & j_{33} \end{pmatrix}. \tag{24}$$

For the model-agnostic refinement the resulting parameters are listed in Table S5.

Table S5: Model-agnostic refinement of the mean-field model for the eight different symmetry-independent j_{xy} for the 3D sample system.

	j_{11}	j_{12}	j_{13}	j_{21}	j_{23}	j_{33}	j_{34}	j_{35}
Neutron								
R (%)	27.3(0)	6.8(2)	23.5(1)	26.9(0)	27.2(0)	26.8(1)	26.8(0)	27.2(0)
βj_{xx}	−0.12(1)	−5.00(0)	0.59(4)	0.59(3)	0.27(2)	−0.22(11)	0.36(2)	−0.23(2)
γ	1.17(0)	0.24(0)	1.00(0)	1.16(0)	1.15(0)	1.16(0)	1.16(1)	1.14(1)
X-ray								
R (%)	18.4(2)	7.0(2)	15.7(1)	21.0(1)	22.2(1)	21.3(1)	22.6(1)	22.5(1)
βj_{xx}	−0.85(12)	−4.91(0)	0.58(6)	1.26(4)	0.27(2)	−0.30(1)	0.26(2)	8.23(0)
γ	8.30(11)	1.71(0)	6.66(1)	7.97(2)	8.24(2)	8.13(0)	-0.19(2)	8.08(2)

DMC simulation driven with the mean-field parameter

As for the 2D case we have driven a DMC simulation with the refined interaction parameter from the mean-field analysis $\beta j = 2.518$ at a Monte Carlo temperature of 1. We used a simulation box of $40 \times 40 \times 40$ unit cells and the resulting configuration correctly matches 98.66% of the tiles.

Warren–Cowley refinement

The diffuse scattering data as refined with the Warren–Cowley approach is compared to the model data in Figure S5. As for the two-dimensional model system the average symmetry dictates most of the pair-correlations and the refined five independent pair-probabilities for the $(1, 0, 0)$ neighbours are listed in Table S6. The refinement resulted in R =11.1(7)% for neutron and R =8.7(4)% for X-ray scattering.

RMC analysis

The RMC analysis uses the same fast Fourier approach for the data calculation as described above with the same rotational averaging of the molecular form factors. The simulation size of the RMC box is $6 \times 6 \times 6$ unit cells, due to the smaller system size $m' = 4$ is chosen for the Lanczos resampling. To save computational time the diffuse scattering for the RMC simulation was calculated on a grid of $161 \times 161 \times 81$ voxels for $-4 \leq h, k \leq 4$ and $-4 \leq l \leq 0$. This volume still represents the full cubic symmetry, as only parts of reciprocal space are omitted that are

Table S6: Warren–Cowley-refined first neighbour $(1, 0, 0)$ pair correlations for X-ray and neutron scattering. 2.8% corresponds to complete random disorder, while 16.6% corresponds to complete order.

pair	Neutron	X-ray
$I \to I$	0.43(26)%	2.54(59)%
$I \to II$	16.14(62)%	13.96(115)%
$II \to I$	2.45(98)%	1.04(62)%
$III \to III$	4.20(18)%	3.49(24)%
$III \to IV$	2.32(31)%	2.64(15)%

Figure S5: Neutron (a) and X-ray (b) diffuse scattering in selected layers for $Hg(NH_3)_2Cl_2$. Top half data as simulated from the model system, bottom half Warren–Cowley refinement.

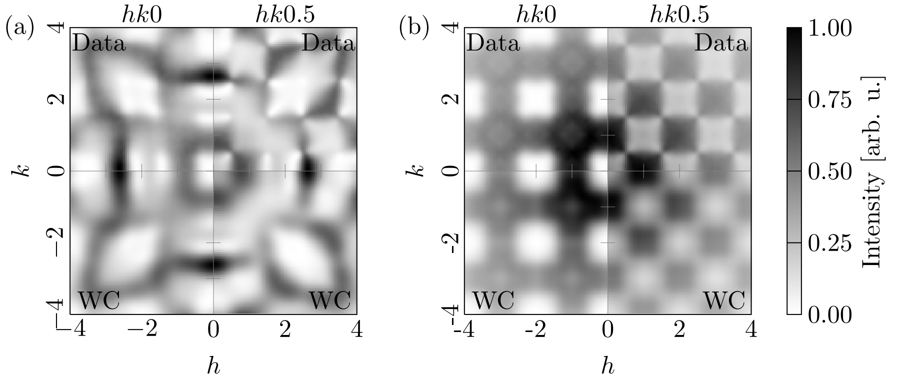

Figure S6: Neutron (a) and X-ray (b) diffuse scattering in selected layers for $Hg(NH_3)_2Cl_2$. Top half data as simulated from the model system, bottom half symmetry-averaged result of RMC refinement.

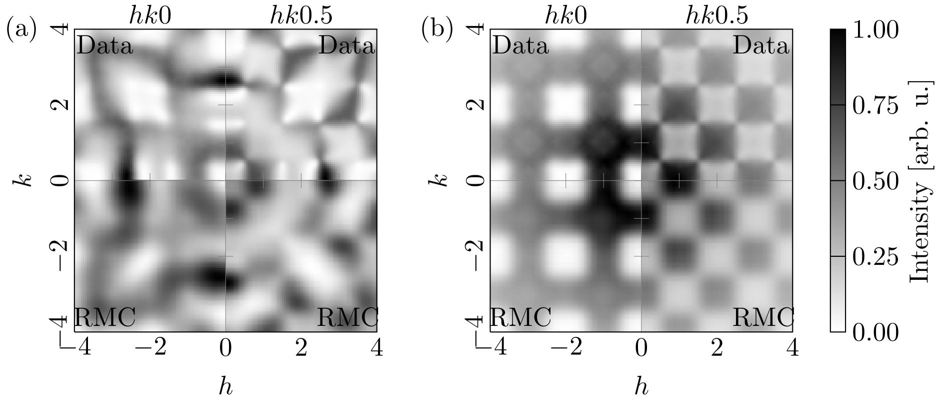

redundant by centro-symmetry.

The RMC simulation was allowed to probe 4320 moves per simulation box which resulted in R-vales of 16.1(2)% before and 15.3(2)% after symmetry averaging for neutron and 8.31(3)% before and 7.36(3)% after symmetry averaging for X-ray scattering. The symmetry-averaged diffuse scattering for a sample refinement is shown in Figure S6.

The resulting symmetry-averaged pair-probabilities for the first shells of neighbours are listed in Table S7 for neutron and in Table S8 for X-ray diffuse scattering. The pair-probabilities, which are not listed, can be derived by symmetry relations.

Table S7: Symmetry-averaged pair probabilities from the RMC analysis for the three-dimensional model system for neutron diffuse scattering. A pair probability of 0.166 corresponds to a completely uncorrelated pair.

vector	I→I	I→II	I→III	I→IV	I→V	I→VI
$\bar{2}00$	0.124(7)	0.188(4)	0.172(6)	0.172(6)	0.172(6)	0.172(6)
$\bar{2}10$	0.164(7)	0.164(4)	0.162(7)	0.170(6)	0.169(6)	0.172(6)
$\bar{2}11$	0.171(4)	0.156(5)	0.169(6)	0.170(5)	0.165(5)	0.168(4)
$\bar{2}2\bar{1}$	0.160(6)	0.166(5)	0.169(7)	0.166(4)	0.169(5)	0.171(6)
$\bar{2}20$	0.171(7)	0.165(7)	0.165(6)	0.164(7)	0.167(6)	0.167(6)
$\bar{2}21$	0.160(6)	0.166(5)	0.166(6)	0.170(5)	0.169(6)	0.168(4)
$\bar{2}22$	0.166(5)	0.167(5)	0.164(5)	0.169(5)	0.165(5)	0.169(6)
$\bar{1}00$	0.064(4)	0.118(7)	0.204(5)	0.204(5)	0.204(5)	0.204(5)
$\bar{1}10$	0.154(4)	0.159(5)	0.160(6)	0.223(5)	0.151(5)	0.152(5)
$\bar{1}11$	0.165(5)	0.172(4)	0.170(4)	0.159(5)	0.178(5)	0.157(5)
$\bar{1}2\bar{1}$	0.164(8)	0.159(6)	0.165(5)	0.175(5)	0.172(6)	0.164(7)
$\bar{1}20$	0.159(7)	0.170(6)	0.162(7)	0.165(6)	0.172(8)	0.173(5)
$\bar{1}21$	0.165(6)	0.159(6)	0.169(6)	0.170(6)	0.165(5)	0.172(7)
$\bar{1}22$	0.163(4)	0.165(3)	0.171(6)	0.166(6)	0.169(6)	0.166(6)
000	1.000(0)	0.000(0)	0.000(0)	0.000(0)	0.000(0)	0.000(0)
010	0.224(4)	0.152(5)	0.204(5)	0.028(3)	0.195(5)	0.197(4)
011	0.153(4)	0.162(5)	0.152(5)	0.191(5)	0.151(5)	0.190(4)
$02\bar{1}$	0.180(8)	0.164(7)	0.169(6)	0.153(5)	0.162(6)	0.173(5)
020	0.143(6)	0.162(5)	0.172(6)	0.195(5)	0.162(4)	0.165(6)
021	0.177(7)	0.164(7)	0.172(6)	0.156(4)	0.172(8)	0.158(6)
022	0.158(6)	0.164(3)	0.167(6)	0.172(5)	0.167(6)	0.171(7)
100	0.064(4)	0.822(3)	0.028(3)	0.028(3)	0.028(3)	0.028(3)
110	0.154(4)	0.228(3)	0.223(5)	0.014(2)	0.191(5)	0.190(4)
111	0.165(5)	0.153(4)	0.157(5)	0.183(5)	0.159(5)	0.183(5)
$12\bar{1}$	0.164(8)	0.179(8)	0.170(5)	0.162(4)	0.154(5)	0.172(7)
120	0.159(7)	0.144(6)	0.170(6)	0.208(5)	0.162(6)	0.158(6)
121	0.165(6)	0.179(8)	0.168(4)	0.157(5)	0.172(6)	0.158(4)
122	0.163(4)	0.158(7)	0.168(4)	0.170(6)	0.169(5)	0.171(6)
200	0.124(7)	0.098(3)	0.195(5)	0.195(5)	0.195(5)	0.195(5)
210	0.164(7)	0.154(4)	0.165(6)	0.208(5)	0.156(4)	0.153(5)
211	0.171(4)	0.165(4)	0.175(5)	0.157(5)	0.170(6)	0.162(4)
$22\bar{1}$	0.160(6)	0.164(7)	0.170(5)	0.170(7)	0.170(6)	0.166(6)
220	0.171(7)	0.153(6)	0.164(7)	0.169(6)	0.172(5)	0.171(7)
221	0.160(6)	0.164(7)	0.166(4)	0.172(6)	0.166(6)	0.171(6)
222	0.166(5)	0.163(5)	0.169(6)	0.167(5)	0.169(5)	0.167(6)

Table S8: Symmetry-averaged pair probabilities from the RMC analysis for the three-dimensional model system for X-ray diffuse scattering. A pair probability of 0.166 corresponds to a completely uncorrelated pair.

vector	I→I	I→II	I→III	I→IV	I→V	I→VI
$\bar{2}00$	0.119(6)	0.158(5)	0.181(5)	0.181(5)	0.181(5)	0.181(5)
$\bar{2}10$	0.167(8)	0.160(4)	0.159(6)	0.180(7)	0.166(6)	0.168(8)
$\bar{2}11$	0.174(9)	0.158(7)	0.165(6)	0.166(6)	0.167(5)	0.170(6)
$\bar{2}2\bar{1}$	0.161(7)	0.166(7)	0.169(5)	0.168(8)	0.168(5)	0.167(6)
$\bar{2}20$	0.170(7)	0.164(6)	0.165(4)	0.164(5)	0.168(5)	0.169(6)
$\bar{2}21$	0.163(5)	0.166(7)	0.166(5)	0.166(6)	0.167(7)	0.173(7)
$\bar{2}22$	0.167(7)	0.166(7)	0.168(6)	0.166(6)	0.163(6)	0.168(7)
$\bar{1}00$	0.320(11)	0.037(2)	0.161(5)	0.161(5)	0.161(5)	0.161(5)
$\bar{1}10$	0.165(6)	0.155(4)	0.141(5)	0.240(7)	0.148(7)	0.151(7)
$\bar{1}11$	0.169(8)	0.169(5)	0.171(7)	0.158(5)	0.174(5)	0.160(6)
$\bar{1}2\bar{1}$	0.168(7)	0.160(7)	0.167(5)	0.170(7)	0.171(7)	0.164(8)
$\bar{1}20$	0.160(7)	0.166(7)	0.159(6)	0.174(5)	0.170(5)	0.171(6)
$\bar{1}21$	0.169(7)	0.160(7)	0.165(6)	0.167(6)	0.163(3)	0.176(7)
$\bar{1}22$	0.164(7)	0.169(5)	0.167(6)	0.165(6)	0.167(7)	0.168(7)
000	1.000(0)	0.000(0)	0.000(0)	0.000(0)	0.000(0)	0.000(0)
010	0.147(4)	0.190(4)	0.161(5)	0.116(6)	0.191(8)	0.195(7)
011	0.154(8)	0.164(5)	0.151(7)	0.190(6)	0.148(7)	0.192(6)
$02\bar{1}$	0.178(7)	0.168(8)	0.166(6)	0.162(5)	0.156(6)	0.171(6)
020	0.151(8)	0.170(8)	0.181(5)	0.179(6)	0.159(4)	0.159(6)
021	0.180(6)	0.168(8)	0.168(8)	0.157(6)	0.170(5)	0.157(7)
022	0.155(5)	0.166(5)	0.169(6)	0.171(7)	0.168(5)	0.171(6)
100	0.320(1)	0.214(3)	0.116(6)	0.116(6)	0.116(6)	0.116(6)
110	0.165(6)	0.175(5)	0.240(7)	0.037(4)	0.190(6)	0.192(6)
111	0.169(8)	0.160(5)	0.160(6)	0.175(7)	0.158(5)	0.178(4)
$12\bar{1}$	0.168(7)	0.186(7)	0.166(6)	0.159(6)	0.146(5)	0.176(7)
120	0.160(7)	0.156(6)	0.180(7)	0.191(5)	0.156(6)	0.157(7)
121	0.169(7)	0.186(7)	0.170(6)	0.161(6)	0.171(7)	0.143(5)
122	0.164(7)	0.157(7)	0.173(7)	0.170(7)	0.168(5)	0.169(7)
200	0.119(6)	0.164(7)	0.179(6)	0.179(6)	0.179(6)	0.179(6)
210	0.167(8)	0.149(3)	0.174(5)	0.191(5)	0.157(6)	0.162(5)
211	0.174(9)	0.170(6)	0.170(7)	0.161(6)	0.167(6)	0.159(6)
$22\bar{1}$	0.161(7)	0.169(7)	0.166(6)	0.166(4)	0.170(7)	0.168(7)
220	0.170(7)	0.149(5)	0.164(5)	0.176(5)	0.171(7)	0.171(6)
221	0.163(5)	0.169(7)	0.168(8)	0.166(5)	0.165(6)	0.169(7)

Refinement times

As for the two-dimensional toy system, we report the total refinement times, including setup calculations, and refinement times per cycle for the Warren–Cowley and mean filed refinement, and the refinement time per move for the RMC refinement in Table S9.

Table S9: Total refinement times, including setup calculations, and refinement times per cycle for Warren–Cowley and mean filed refinement, and the refinement time per move for the RMC refinement for the 3D system.

	mean-field		Warren–Cowley		RMC	
	Neutron	X-ray	Neutron	X-ray	Neutron	X-ray
Total time (s)	517.4	636.0	2377.7	1208.4	3822.2	4647.2
Time per cycle/move (s)	4.23	4.16	3.42	3.52	0.87	1.11

4 Stability against missing data

Supplementary data for the 2D model system

Mean-field refinement

The mean-field refinement yielded the parameters as shown in Table S10. For both X-ray and neutron scattering data, the mean-field refinement gives very similar results to the complete data set refinement when fitting only a 10 degree wedge of the data or punching the data around the Bragg reflections. The refinement for the one-dimensional $h0$-cut is less stable and leads to larger uncertainties in the refinement.

Table S10: Parameters of the mean-field analysis in the two-dimensional model system with limited data ranges. R-values are only evaluated on the given section of the data. The missing data input is shown in Figure 7 of the main text.

	neutron			X-ray		
	Bragg	$h0$	10 Deg.	Bragg	$h0$	10 Deg.
βj	1.669(6)	1.3(5)	1.527(12)	1.54(10)	0.4(5)	0.9(2)
Scale	3.83(6)	9(3)	5.3(12)	5.2(11)	20.0(10)	13(10)
R (%)	16.4(9)	11(6)	14(7)	19.6(11)	14(2)	21.5(9)

RMC refinement

The resulting correlations of the RMC analysis with missing data are visualized in Figure S7 with the same colour scale as used in Figure 3(c) of the main text. The correlations clearly show that the RMC approach fails to identify the local ordering principle for all attempts for the X-ray scattering, while for neutron scattering it seems to be relatively robust against missing data around the Bragg reflections.

Figure S7: Symmetry-averaged correlations of the RMC analysis with missing data. The occupation of the mercury is depicted indicated by the identical grey scale to Figure 3 of the main text and the input data is the same as shown in Figure 7 of the main text. (a,c,d) Neutron. (b,e,f) X-ray.

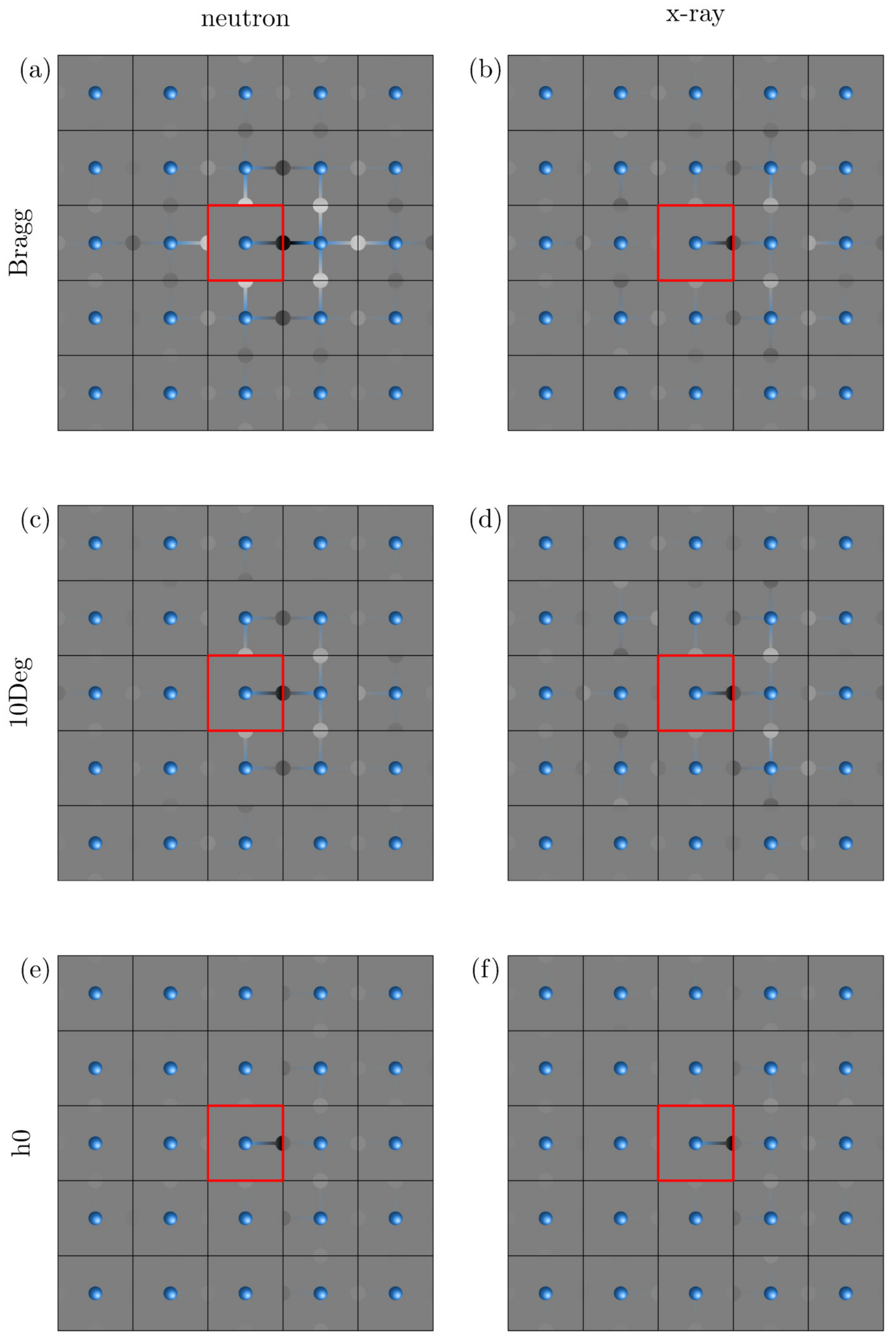

Warren–Cowley refinement

The pair-probabilities as refined by the Warren–Cowley approach are listed in Table S11. In a similar fashion to the mean-field refinement, the Warren–Cowley approach can identify the dominating local pair correlation. The resulting R-values are also listed in Table S11.

Table S11: Warren–Cowley refined first neighbour $(1, 0)$ pair correlations for neutron and X-ray scattering and missing data sets. The last row gives the resulting R-values of the refinements.

	Neutron			X-ray		
pair	Bragg	10deg	1D	Bragg	10deg	1D
$I \to I$	2.36(12)%	5.12(99)%	5.85(307)%	2.67(83)%	4.17(17)%	1.22(19)%
$I \to II$	6.77(07)%	7.34(18)%	8.01(389)%	9.40(79)%	7.29(22)%	8.05(18)%
$II \to I$	0.47(03)%	0.02(13)%	0.02(5)%	0.37(19)%	0.01(7)%	3.91(12)%
$II \to II$	17.19(31)%	10.22(105)%	8.73(182)%	15.50(82)%	15.51(55)%	3.53(21)%
$III \to I$	21.68(1)%	19.839(118)%	19.11(305)%	22.24(96)%	20.80(23)%	17.04(27)%
R	22.72(2)%	11.5(4)%	11.1(4)%	12.6(6)%	13.1(5)%	5.9(5)%

Model-agnostic refinement for 2D missing data

For the two-dimensional system and the restricted data input it was also tried to run the model-agnostic refinement. The results are presented in Figure S8. For all situations that are presented the fit with j_{13} yields significantly better results than with the other j_{xy}. Hence, even with severely limited data coverage the mean-field approach is still capable of identifying the correct pair-interaction model.

Figure S8: Model-agnostic refinement for the two-dimensional models system and missing data input. The labels of the interaction parameters j_{xy} correspond to the labels of Equation (10) of the main text. Neutron (N) scattering in blue, X-ray (X) scattering in magenta.

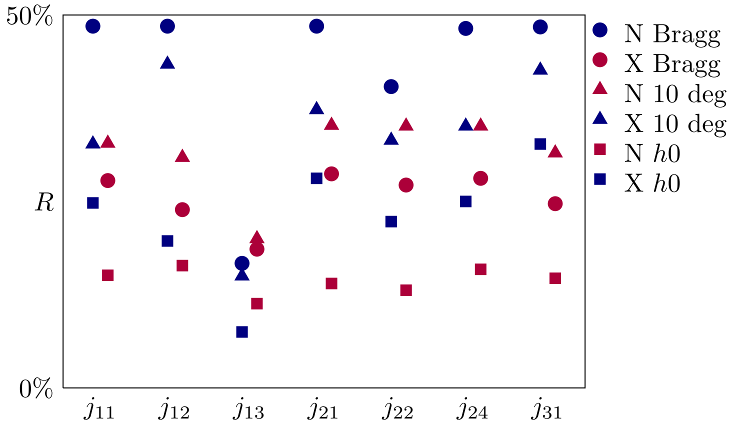

Missing data in 3D refinements

In a very similar fashion to the 2D refinements with missing data discussed in the main text, the 3D data were refined with the mean-field approach and the pair-interaction Hamiltonian in Equation 16. Four different situations were tested:

1. A chunk around the Bragg reflections was missing.
2. A 10 degree cone was taken out of the data.
3. Only the $hk0$-layer was provided.
4. Only the $hk0.5$-layer was provided.

The results for this refinement approach are listed in Table S12. The results reproduce the results from the two-dimensional model system: The mean-field approach is able to reliably fit the interaction model, even when the data is restricted to one layer in reciprocal space. This suggests that the approach as presented here could *e. g.* be exploited to analyse zone axis patterns from electron diffraction experiments.

Table S12: R-values, βj and γ refined with the mean-field approach and the pair-interaction Hamiltonian in Equation 16 for the three dimensional case with missing data.

	Neutron			X-ray		
	R (%)	βj	γ	R (%)	βj	γ
Bragg	6.77(22)	2.52(3)	2.43(1)	6.48(23)	2.45(0)	2.77(2)
10 deg cone	6.98(20)	2.60(6)	2.10(3)	7.17(3)	2.47(0)	2.78(0)
$hk0$-layer	6.20(33)	3.39(1)	2.17(0)	4.45(40)	2.12(1)	4.66(1)
$hk0.5$-layer	8.86(45)	2.63(0)	1.78(2)	8.76(43)	2.52(1)	2.32(4)

5 References

(S1) Neder, R. B. & Proffen, T. (2008). *Diffuse Scattering and Defect Structure Simulations*, vol. 11. Oxford University Press.

(S2) Paddison, J. A. M. (2019). *Acta Cryst.* **A75**(1), 14–24.

(S3) Overy, A. R., Cairns, A. B., Cliffe, M. J., Simonov, A., Tucker, M. G. & Goodwin, A. L. (2016) *Nat. Commun.* **7**, 10445.

(S4) Withers, R. L., Welberry, T. R., Larsson, A.-K., Liu, Y., Norén, L., Rundlöf, H. & Brink, F. J. (2004) *J. Solid State Chem.* **177**, 231-244.
